# Degradable water-soluble polymer prodrugs for subcutaneous delivery of irritant anticancer drugs†

**DOI:** 10.1039/d5sc02967h

**Published:** 2025-07-10

**Authors:** Léa Guerassimoff, Jingming Cao, Michaella Auguste, Amaury Bossion, Chen Zhu, Dao Le, Catherine Cailleau, Safa Mohamed Ismail, Françoise Mercier-Nomé, Julien Nicolas

**Affiliations:** a Université Paris-Saclay, CNRS, Institut Galien Paris-Saclay 91400 Orsay France julien.nicolas@universite-paris-saclay.fr +33 1 80 00 60 81; b Université Paris-Saclay, IPSIT, INSERM UMR 996 91400 Orsay France

## Abstract

Chemotherapy is primarily administered intravenously (IV), but this route poses significant challenges (*e.g.*, high costs, patient discomfort, logistical difficulties, side effects such as infections from catheter use). Although oral and subcutaneous (SC) routes are preferred for their convenience and have the potential for better patient comfort and cost reduction, oral chemotherapy faces issues like poor bioavailability and adherence, while SC delivery is unsuitable for irritant or vesicant drugs due to local toxicity. To overcome these limitations, the polymer prodrug strategy has been explored, where drugs are linked to a polymer, reducing toxicity and enhancing drug delivery. Recent work has focused on creating water-soluble polymer prodrugs for SC delivery of paclitaxel (Ptx), a hydrophobic and vesicant drug, which was successfully conjugated to polyacrylamide (PAAm), a very hydrophilic biocompatible polymer, resulting in safer SC injection and enhanced therapeutic efficacy in tumor-bearing mice. However, this strategy's potential depends on adapting it to other vesicant anticancer drugs. Making the polymer degradable for facilitated excretion would also be a key improvement. In this work, this approach has been successfully extended to gemcitabine (Gem), a widely used but irritant anticancer drug, and to a degradable PAAm-based promoiety, having cleavable ester groups in the main chain. The resulting Gem-based prodrugs featured upper critical solution temperature to ensure complete solubility at the temperature of the SC tissue, sustained Gem release, significant degradation under physiological conditions, improved systemic toxicity and absence of local toxicity compared to free Gem. Remarkably, Gem-PAAm polymer prodrugs exhibited significant anticancer efficacy in mice bearing Mia Pa-Ca 2 tumors, outperforming Gemzar®, the commercial formulation of Gem. These advances suggest the potential of these hydrophilic polymer prodrugs to transform SC chemotherapy, enabling the use of a broader range of anticancer drugs while reducing side effects and improving patient outcomes.

## Abstract 2

Degradable, water-soluble polymer prodrugs based on gemcitabine were obtained by rROP. They exhibited no local toxicity after subcutaneous injection to mice and their non-degradable counterparts outperformed Gemzar in a pancreatic tumor mouse model.

## Graphical abstract

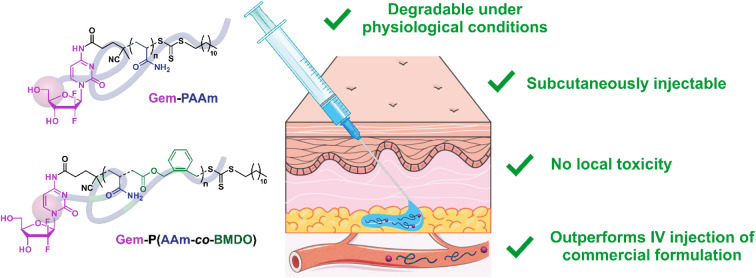

## 1. Introduction

Chemotherapies are almost exclusively administered by the intravenous (IV) route.^1^ Nonetheless, this route poses a number of major problems in terms of logistics, patient comfort and cost. For example, the high cost of IV chemotherapy comes from outpatient hospital visits, surgical interventions and long hospital stays due to repeated administration cycles or long infusions.^2^ The IV route is also restrictive for the patient who must be hospitalized regularly, and is invasive due to the need for a central catheter and an implantable chamber, which often leads to serious side effects (*e.g.*, infections),^3,4^ further prolonging hospital stays and increasing costs.^5^ Although the oral and subcutaneous (SC) routes are preferred, notably for their ease of use,^6–8^ oral delivery suffers from poor and variable bioavailability, as well as compliance problems. The SC route is also unsuitable for many vesicant/irritant drugs because of their local toxicity^9^ at the injection site,^10^ such as skin reactions (*e.g.*, alopecia, hyperpigmentation, irritation, necrosis) as well as SC cell dysfunction and death after repeated injections.^11^ This is unfortunate as SC administration could offer an ideal compromise due to its high bioavailability and rapid drug absorption,^12^ while being able to be implemented at home, resulting in lower costs and greater comfort for the patient.^13^

Although a wide range of nanoscale drug delivery systems have been developed for SC administration,^14^ those dedicated to cancer therapy are limited to non-irritant and non-vesicant anticancer drugs and biomacromolecules (*e.g.*, therapeutic proteins, antibodies). To alleviate these limitations, it is possible to take advantage of the polymer prodrug approach, which involves coupling drugs to a polymer scaffold *via* a cleavable linker, resulting in transient drug inactivation.^15–19^ In this context, we have recently reported the design and the preclinical evaluation of water-soluble polymer prodrugs suitable for the SC delivery of vesicant and irritant anticancer drugs.^20^ The principle is based on the “drug-initiated” synthesis of well-controlled polyacrylamide (PAAm) chains carrying one drug molecule at the chain end through the use of a drug-bearing controlling agent to perform controlled radical polymerization of AAm. PAAm was selected for its high water-solubility, biocompatibility and stealth properties,^21^ and we applied this approach to paclitaxel (Ptx), a highly hydrophobic and vesicant anticancer drug used in the formulation of Taxol, as a proof to validate our strategy. The obtained Ptx-PAAm prodrugs enabled safe SC injection without inducing local toxicity and outperformed Taxol during efficacy study in tumor-bearing mice, due to a higher maximum tolerated dose (MTD).^20^

However, the robustness and the versatility of this strategy, and therefore its future potential for drug delivery, depend primarily on its applicability to other irritant/vesicant drugs. Indeed, transforming this approach into a SC drug delivery platform by adapting it to other anticancer drugs could considerably broaden its field of application and pave the way for the treatment of different types of cancer. In addition, making the polymer promoiety degradable under physiological conditions to facilitate excretion and prevent accumulation in the body would undoubtedly be a key improvement with a view to possible clinical transposition.

**Fig. 1:**
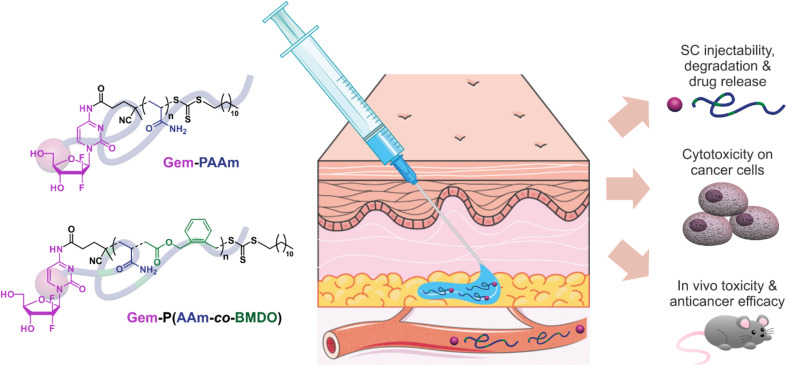
Design and preclinical development of (degradable) polyacrylamide (PAAm)-based prodrugs for the SC administration of the anticancer drug gemcitabine (Gem).

Herein, we addressed both challenges by developing PAAm prodrugs for SC administration based on the irritant anticancer drug gemcitabine (Gem) and by making these prodrugs degradable under physiological conditions (Fig. 1).^9,22,23^ Gem is a nucleoside analogue with proven activity against a broad range of solid tumors (*e.g.*, colon, lung, pancreatic, breast, bladder, ovarian cancers).^24^ However, like many nucleoside analogues, Gem suffers from serious limitations that often restrict its use, such as its irritant nature, short plasma half-life, rapid metabolism (due to deamination), induction of resistance, and the advent of severe side effects.^25^ A polymer prodrug strategy based on Gem would therefore not only avoid local toxicity after SC injection, but also prolong its half-life time by avoiding early degradation and improve therapeutic efficacy. As for the degradable nature of Gem-based polymer prodrugs, it was achieved by inserting ester groups in the PAAm backbone by controlled radical ring-opening polymerization (rROP).^26–30^ More specifically, we copolymerized AAm and 5,6-benzo-2-methylene-1,3-dioxepane (BMDO) as a cyclic ketene acetal (CKA) monomer, during the “drug-initiated” synthesis of the polymer prodrug. Interestingly, the resulting Gem-P(AAm-*co*-BMDO) copolymers exhibited an upper critical solution temperature (UCST),^31^ governed by an equilibrium between polymer–polymer interactions and polymer–aqueous medium interactions,^32^ which was finely tuned to produce fully water-soluble polymer prodrugs at SC tissue temperature (33–35 °C),^33^ thus preventing early degradation of the copolymer and drug release. Gem-P(AAm-*co*-BMDO) copolymer prodrugs demonstrated sustained release of Gem in human serum, significant *in vitro* cytotoxicity on a pancreatic cancer cell line and did not induce any local or systemic adverse effect at high doses after SC injection to mice, conversely to free Gem. These innovative prodrugs also resulted in higher survival rates and greater anticancer efficacy in tumor-bearing mice, compared with IV injection of Gemzar, the commercial formulation of Gem.

## 2. Experimental part

### 2.1 Materials

Azobisisobutyronitrile (98%, AIBN) and acrylamide (>99%, AAm) were purchased from Sigma-Aldrich and recrystallized from ethanol and chloroform, respectively. 1-(3-Dimethylaminopropyl)-3-ethylcarbodiimide hydrochloride (>97%, EDC·HCl), *N*-hydroxysuccinimide (98%, NHS), cyano-4-[(dodecylsulfanylthiocarbonyl) sulfanyl]pentanoic acid (97%, CDP), anhydrous dimethylsulfoxide (≥99.9%, DMSO), anhydrous dimethylformamide (≥99.9%, DMF), triethylamine (≥99%, TEA), anhydrous theophylline (≥99%), tetrahydrouridine (THU), potassium hydroxide (90%, KOH), Dulbecco's Phosphate Buffer Saline (PBS), Dulbecco's Modified Eagle Medium (DMEM) and human serum were purchased from Sigma-Aldrich and used as received. Gemcitabine·HCl (>98%, Gem) was purchased from TCI (Europe). Deuterated DMSO (DMSO-*d*_6_) was obtained from Eurisotop. Methanol (HPLC analytical grade, MeOH) and diethyl ether (HPLC analytical grade) were purchased from Carlo Erba. All other solvents were purchased from Sigma-Aldrich at the highest grade. ROTI®Histofix 4% (formaldehyde, pH 7) was purchased from Roth.

### 2.2 Analytical methods

#### 2.2.1 Nuclear magnetic resonance (NMR) spectroscopy

NMR spectroscopy was performed in 5 mm diameter tubes in DMSO-*d*_6_ at 25 °C. ^1^H-NMR spectroscopy was performed on a Bruker Avance 300 spectrometer at 300 MHz and on a Bruker Avance 400 at 400 MHz. The chemical shift scale was calibrated based on the internal solvent signals (*δ* = 2.50 ppm for DMSO-*d*_6_). ^13^C-NMR spectroscopy was performed on a Bruker Avance 400 at 100 MHz. ^19^F-NMR spectra were recorded on a Bruker Avance 400 at 376.5 MHz. Data were processed with MestReNova 14.0.0 software.

#### 2.2.2 Mass spectrometry

Mass spectra were recorded with a Bruker Esquire-LC instrument. High-resolution mass spectra (ESI) were recorded on an ESI/TOF (LCT, Waters) LC-spectrometer.

#### 2.2.3 Size exclusion chromatography (SEC)

SEC was performed at 60 °C using two columns in series from Agilent Technologies (PL PolarGel-M, 300 × 7.5 mm; bead diameter 8 μm; molar mass range 1000–500 000 g mol^−1^) preceded by a guard column from Agilent Technologies (PL PolarGel-M, 7.5 × 50 mm; bead diameter 8 μm) and a triple detection system (Viscotek TDA/GPCmax from Malvern) with a differential refractive index detector, low and right-angle light scattering detectors and a differential viscometer detector. The eluent was DMSO with 100 mM lithium bromide (LiBr) and 0.4 wt% of 2,6-di-*tert*-butyl-4-methylphenol (BHT) as a flowrate marker at a flow rate of 0.7 mL min^−1^. The system was calibrated using poly(methyl methacrylate) (PMMA) standards (peak molar masses, *M*_p_ = 540–342 900 g mol^−1^) from Agilent Technologies. This allowed the determination of the number-average molar mass (*M*_n_), the weight-average molar mass (*M*_w_) and the dispersity (*Đ* = *M*_w_/*M*_n_). All samples were filtered over 0.22 μm Nylon filters prior to injection. Data were collected and processed with OmniSEC 4.0 software.

#### 2.2.4 UV-Vis spectroscopy

Light transmittance was measured using a Lambda 25 UV/VIS spectrometer equipped with a PTP 1 + 1 Peltier system for temperature control (PerkinElmer) at a wavelength of 500 nm, with a cell path length of 10 mm and under magnetic stirring. Samples were prepared at different concentrations in MilliQ water and placed in a quartz cuvette. The measurements were carried out by first cooling the solution from the lowest temperature (*T* ≪ UCST) at a constant rate of 1 °C min^−1^, followed by heating the solution back to the starting temperature at the same rate. The inflection point of the transmittance curve was considered as the UCST cloud point. It was graphically determined by the maximum of the first derivative of the cooling/heating curves. Data were collected with Winlab 6.0.3.0730 software and processed with Data Processor and Viewer (DPV) 1.00.100.0010 software.

#### 2.2.5 Dynamic light scattering (DLS)

The nanoparticle intensity-weighted and number-weighted mean diameters (*D*_z_ and *D*_n_, respectively) were measured by DLS with a Nano ZS Ultra from Malvern equipped with a 4–10 mW He–Ne laser (633 nm wavelength) at three different measuring angles of 173, 13 and 90°. Similar to UV-Vis measurements, samples were prepared at different concentrations in MilliQ water and placed in a quartz cuvette. The cooling and heating measurements were taken at an interval of 1 °C starting from *T* ≪ UCST (minimum 1 °C) and the solution was equilibrated at each temperature for 120 s prior to measurements. The inflection point of the intensity- or number-average diameters curve was considered to be the *T*_cp_ value. Data were processed with Malvern-Zetasizer 1.5.0.163 software.

#### 2.2.6 Injectability

Injectability tests were carried out using a custom-made device as described elsewhere.^34^ This device was coupled to a texture analyzer TAXT2 (Stable MicroSystems, Godalming, UK) in compression mode, which was equipped with a force transducer calibrated with a 30 kg sensor. 400 μL of solution was taken in a 1 mL syringe (MeritMedical, Medaillon Syringe, USA), which was then fitted with a 26 G × ½′′ needle (0.45 × 12 mm, Terumo Neolus, Japan) before injection at a 1 mm s^−1^ test speed. Each measurement was repeated 6 times for each sample.

### 2.3 Synthesis procedures

#### 2.3.1 Synthesis of Gem-CDP RAFT agent

In a round-bottom flask, CDP (808.3 mg, 2 mmol), EDC·HCl (767.8 mg, 4 mmol) and NHS (240.3 mg, 2.4 mmol) were dissolved into dry DMF (25 mL). The mixture was stirred during 2 h at room temperature. In a second round-bottom flask, Gem (600 mg, 2 mmol) was dissolved with TEA (0.28 mL, 2 mmol) into dry DMF (25 mL). After 2 h, the solution of Gem/TEA was added dropwise into the reaction mixture and stirred at RT for 24 h. After 24 h, the reaction was stopped and the mixture was then diluted into ethyl acetate (90 mL). The product is then washed with 10% HCl (60 mL), saturated NaHCO_3_ solution (60 mL) and brine NaCl (60 mL), before drying over MgSO_4_. The organic phase is finally evaporated and concentrated under vacuum to give the Gem-CDP RAFT agent as a yellow powder. The crude mixture was purified by column chromatography (dichloromethane (DCM) : MeOH 20 : 1) to give 170 mg of a yellow oil (30% yield). ^1^H-NMR (400 MHz, DMSO-*d*_6_) *δ* 11.10 (s, 1H), 8.25 (d, *J* = 7.6 Hz, 1H), 7.24 (d, *J* = 7.6 Hz, 1H), 6.29 (d, *J* = 6.5 Hz, 1H), 6.18 (t, *J* = 7.5 Hz, 1H), 5.26 (t, *J* = 5.3 Hz, 1H), 4.18 (m, *J* = 8.1 Hz, 1H), 3.89 (m, *J* = 8.0 Hz, 1H), 3.83–3.61 (m, 2H), 3.42–3.35 (t, 2H), 1.86 (s, 2H), 1.68–1.60 (m, 2H), 1.24 (s, 16H), 0.86 (t, *J* = 6.5 Hz, 3H). ^19^F-NMR (400 MHz, DMSO-*d*_6_) *δ* ppm: 116.7–117.1 (s, 1F). ^13^C-NMR (100 MHz, DMSO-*d*_6_) *δ* 218.26, 173.04, 171.96, 162.72, 154.16, 144.53, 122.82, 118.51, 95.73, 84.00, 80.76, 58.66, 36.40, 32.65, 31.84, 31.27, 28.93, 28.86, 28.69, 28.59, 28.36, 28.06, 27.24, 25.22, 23.86, 22.07, 13.86. High-resolution mass spectrometry (ESI^+^): *m*/*z* = [M + H^+^]. Calculation = 648.299, found = 649.237.

#### 2.3.2 Synthesis of Gem-P(AAm-*co*-BMDO) copolymer prodrugs (P0–P4)

Gem-P(AAm-*co*-BMDO) copolymer prodrugs were synthesized from Gem-CDP as a RAFT agent. Different initial molar ratios of AAm and BMDO were tested (50 : 50 (P0), 55 : 45 (P1), 56 : 44 (P2), 57 : 43 (P3) and 60 : 40 (P4), respectively). For Gem-P(AAm-*co*-BMDO) with a AAm : BMDO molar ratio of 56 : 44 (P2), AAm (112 eq., 4.48 mmol, 0.32 g), BMDO (88 eq., 3.52 mmol, 0.57 g) (total mole = 8 mmol), Gem-CDP (1 eq., 0.04 mmol, 26 mg) and AIBN (0.6 eq., 0.024 mmol, 3.9 mg) were dissolved in anhydrous DMSO (10 mL). The solution was bubbled with dry argon to remove dissolved oxygen for 20 min at room temperature and then immersed in a preheated oil bath at 70 °C for 16 h. The solution was then rapidly cooled under air. The copolymer was precipitated into cold methanol and then washed three times with cold methanol followed by centrifugation (5000 rpm, 10 min). The copolymer prodrug was then dried under high vacuum to give 179 mg of a white powder. The purified copolymer prodrugs were characterized by NMR and SEC. ^1^H-NMR (400 MHz, DMSO-*d*_6_) *δ* ppm: 0.82–0.88 (t, 3H–C_12_), 1.22–1.25 (s, H–C_12_), 1.27–1.54 (m, 2H-AAm), 2.00–2.27 (m, 1H-AAm), 2.68–2.71 (d; 2H-BMDO), 3.76–3.93 (m, 2H-Gem), 4.43–4.74 (m, 4H-closed BMDO), 4.93–5.17 (m, 2H-opened BMDO), 5.21–5.35 (m, 1H-Gem), 6.14–6.33 (m, 1H-Gem), 6.66–7.45 (m, 2H-AAm + 4H-BMDO + 1H-Gem), 8.12–8.27 (dd, 2H Gem),11.03–11.12 (t, 1H-Gem).^19^F-NMR (400 MHz, DMSO-*d*_6_) *δ* ppm: 116.7–117.1 (s, 1F). The same procedure was carried out for other copolymer prodrugs, except that P4 was precipitated into cold THF and P0–P3 were precipitated into cold methanol due to different amounts of BMDO. Note that P0 and P4 were analyzed by ^1^H-NMR (300 MHz, DMSO-*d*_6_).

#### 2.3.3 Synthesis of P(AAm-*co*-BMDO) copolymers (P5–P6)

P(AAm-*co*-BMDO) were synthesized following a previously developed procedure^31^ and adapted as follows. Two initial AAm : BMDO molar ratios (56 : 44 and 57 : 43) were tested. For P(AAm-*co*-BMDO) 56 : 44 (P5), AAm (112 eq., 4.48 mmol, 0.32 g), BMDO (88 eq., 3.52 mmol, 0.57 g) (total mole = 8 mmol), CDP (1 eq., 0.04 mmol, 16.1 mg) and AIBN (0.6 eq., 0.024 mmol, 3.9 mg) were dissolved into anhydrous DMSO (10 mL). The solution was bubbled with dry argon to remove dissolved oxygen for 20 min at room temperature and then immersed in a preheated oil bath at 70 °C for 16 h. The solution was then rapidly cooled under air. The copolymer was precipitated into cold methanol and then washed three times with cold methanol followed by centrifugation (5000 rpm, 10 min). The copolymer was then dried under high vacuum to give 185 mg of a white powder. The purified copolymers were characterized by ^1^H-NMR and SEC. ^1^H-NMR (300 MHz, DMSO-*d*_6_) *δ* ppm: 0.82–0.89 (t, 3H–C_12_), 1.20–1.31 (m, H–C_12_), 1.31–1.73 (m, 2H-AAm), 2.01–2.25 (m, 1H-AAm), 4.55–4.77 (m, 4H-closed BMDO), 4.97–5.21 (m, 2H-opened BMDO), 6.59–7.52 (m, 6H: 2H-AAm + 4H-BMDO).

#### 2.3.4 Synthesis of Gem-PAAm polymer prodrug (P7)

Gem-PAAm P7 was synthesized as follows. A mixture of AAm (130 eq., 15 mmol, 1.07 g) (total mole = 15 mmol), Gem-CDP (1 eq., 0.115 mmol, 74.9 mg) and AIBN (0.6 eq., 0.069 mmol, 11.4 mg) was dissolved in anhydrous DMSO (12 mL). The solution was bubbled with dry argon to remove dissolved oxygen for 20 min at room temperature and then immersed in a preheated oil bath at 70 °C for 16 h. The solution was then rapidly cooled under air. The polymer prodrug was precipitated into cold THF and then washed three times with cold THF followed by centrifugation (5000 rpm, 10 min). To remove residual traces of unreacted AAm, the polymer prodrug was then precipitated into acetone and then washed three times with acetone under Büchner filtration. The obtained Gem-PAAm prodrug was then dried under high vacuum until to give 1.03 g of a white powder The purified polymer prodrug was characterized by NMR and SEC. ^1^H-NMR (400 MHz, DMSO-*d*_6_) *δ* ppm: 0.82–0.88 (t, 3H–C_12_), 1.22–1.25 (s, H–C_12_), 1.30–1.57 (m, 2H-AAm), 1.96–2.25 (m, 1H-AAm), 3.76–3.93 (m, 2H-Gem), 5.28–5.33 (m, 1H-Gem), 6.14–6.21 (t, 1H-Gem), 6.58–7.38 (m, 2H-AAm + 1H-Gem), 7.46–7.63 (d, 1H, Gem), 8.22–8.26 (d, 1H Gem),11.04–11.12 (t, 1H-Gem). ^19^F-NMR (400 MHz, DMSO-*d*_6_) *δ* ppm: 116.7–117.1 (s, 1F).

#### 2.3.5 Synthesis of PAAm polymer (P8)

PAAm was synthesized as follows. A mixture of AAm (135 eq., 4 mmol, 0.28 g) (total mole = 4 mmol), CDP (1 eq., 0.03 mmol, 11.9 mg) and AIBN (0.6 eq., 0.018 mmol, 2.9 mg) was dissolved in anhydrous DMSO (3 mL). The solution was bubbled with dry argon to remove dissolved oxygen for 20 min at room temperature and then immersed in a preheated oil bath at 70 °C overnight. The solution was then rapidly cooled under air. The polymer was precipitated into cold THF and then washed three times using cold volumes of THF and centrifugations (5000 rpm, 10 min). PAAm obtained was then dried under high vacuum until to give 310 mg of white powder. The purified polymer was characterized by ^1^H-NMR and SEC. ^1^H-NMR (400 MHz, DMSO-*d*_6_) *δ* ppm: 0.83–0.89 (t, 3H–C_12_), 1.24–1.36 (s, H–C_12_), 1.31–1.65 (m, 2H), 2.02–2.22 (s, 1H), 6.60–7.36 (dd, 2H).

#### 2.3.6 Synthesis of Gem-PAAm polymer prodrugs for anticancer efficacy studies (P9–P11)

Gem-PAAm with three different targeted chain lengths [*M*_n,th_ = 5000 g mol^−1^ (P9), 10 000 g mol^−1^ (P10) and 20 000 g mol^−1^ (P11)] were synthesized by varying the initial amount of AAm, the amounts of the other reagents being fixed (AAm = 0.63, 1.26 and 2.53 g, for P9–P11, respectively). For P9, the synthesis was as follows: AAm (78 eq., 8.91 mmol, 0.63 g), Gem-CDP (1 eq., 0.115 mmol, 74.9 mg) and AIBN (0.6 eq., 0.069 mmol, 11.4 mg) were dissolved in anhydrous DMSO (6 mL). The solution was bubbled with dry argon to remove dissolved oxygen for 20 min at room temperature and then immersed in a preheated oil bath at 70 °C for 16 h. The solution was then rapidly cooled under air. The polymer was precipitated into cold methanol and then washed three times with cold methanol followed by centrifugation (5000 rpm, 10 min). It was then dried under high vacuum to give 505 mg of a white powder. The purified polymer was characterized by ^1^H-NMR and SEC. ^1^H-NMR (400 MHz, DMSO-*d*_6_) *δ* ppm: 0.82–0.88 (t, 3H–C_12_), 1.18–1.28 (s, H–C_12_), 1.30–1.73 (m, 2H-AAm), 1.96–2.27 (m, 1H-AAm), 3.57–3.71(m, 1H-Gem), 3.76–3.93 (m, 2H-Gem), 4.06–4.25 (m, 1H-Gem), 5.21–5.35 (m, 1H-Gem), 6.15–6.20 (t, 1H-Gem), 6.30–6.32 (d, 1H-Gem), 6.41–7.65 (m, 2H-AAm), 8.23–8.25 (d, 1H Gem), 11.01–11.13 (t, 1H-Gem). The same procedure was followed for P10 and P11, yielding 1.1 g of P10 and 1.3 g of P11 after drying under high vacuum.

### 2.4 Degradation procedures

#### 2.4.1 Accelerated hydrolytic degradation

25 mg of copolymer prodrugs (P0–P4) were dissolved in aqueous KOH solution (1.25 mL, 5 wt%). The mixture was stirred for 1 h at room temperature, after which the solution became completely transparent. The solution was then quenched by adding an aqueous solution of HCl 1 M (1 mL). The resulting solution was freeze-dried overnight to give a white powder. The degraded products were characterized by SEC.

#### 2.4.2 Hydrolytic degradation under physiological conditions

20 mg of copolymer prodrugs (P1–P3) were solubilized into PBS (2 mL, pH 7.4) and stirred under a thermostated orbital shaker (IKA KS4000i control) at 100 rpm and 37 °C. At specific time intervals (*i.e.*, days 1, 3, and 7), samples of 0.5 mL were withdrawn and freeze-dried to give a white powder. The degraded products were characterized by SEC.

### 2.5 *In vitro* evaluations

#### 2.5.1 Drug release monitored by HPLC

Gem release was monitored following a previously published procedure^35^ and adapted as follows. 0.2 mL of nanoparticle suspension were added to 0.8 mL of human serum supplemented with 200 μg per mL tetrahydrouridine (THU).^36,37^ The mixture was aliquoted (100 μL), incubated at 37 °C, withdrawn at different time points (1, 2, 4, 6, 8, 24, 48 and 168 h) and spiked with 10 μL of 10 μM theophylline before addition of 1 mL of a mixture of acetonitrile : methanol (90 : 10, v/v), followed by ultracentrifugation (13 200 rpm, 20 min). The supernatant was then evaporated to dryness under a nitrogen flow at 30 °C and the released drug was quantified by reverse-phase HPLC. The chromatographic system was composed of a Waters 1525 Binary HPLC pump, a Waters 2707 Autosampler, a C18 Uptisphere column (3 μm, 150 × 4.6 mm; Interchim), HPLC column temperature controllers (model 7950 column heater and chiller; Jones Chromatography, Lakewood, CO) and a Waters 2998 programmable photodiode-array detector. The HPLC column was maintained at 30 °C and detection was monitored at 270 nm. The HPLC mobile phase was a mixture of methanol:water with 0.05 M sodium acetate (pH 5.0, eluent A: 5 : 95, v/v; eluent B: 97 : 3, v/v). The residues were dissolved in 100 μL of eluent A and centrifuged (13 200 rpm, 5 min) before analysis. Elution was performed at a flow rate of 0.8 mL min^−1^ isocratically for 8 min with eluent A followed by a linear gradient (1 min) to 75% eluent A and kept isocratically for 6 min at 75% eluent A. A linear gradient (1 min) to 100% eluent B was followed by 10 min of isocratic gradient at 100% eluent B. After a linear gradient (1 min) to 100% eluent A, the system was held for 7 min for equilibration back to initial conditions. HPLC graphs and calibration curves can be found in Fig. S1–3,† respectively.

#### 2.5.2 Cell lines and cell culture

Human pancreatic cancer cell line Mia PaCa-2 was obtained from the American Type Culture Collection and maintained as recommended. Briefly, Mia PaCa-2 cells were grown in Dulbecco's minimal essential medium (DMEM) with 10% fetal bovine serum, penicillin (100 U mL^−1^), streptomycin (100 U mL^−1^) and 2.5% horse serum (Gibco). Cells were maintained in a humid atmosphere at 37 °C with 5% CO_2_.

#### 2.5.3 Cell viability assay

Gem-P(AAm-*co*-BMDO) copolymer prodrugs (P2 and P3) and drug-free copolymers P(AAm-*co*-BMDO) (P5 and P6) solutions were heated at *T* > UCST to maintain a solubilized state before use. Gem-PAAm P7 and PAAm P8 were solubilized into MilliQ water to achieve desired concentrations. In 96-well microtiter plates (TPP, Switzerland), cells were seeded (5 × 10^3^ cells per mL) in 100 μL of growth medium and pre-incubated for 24 h in incubator (37 °C and 5% CO_2_). After appropriate dilutions, 100 μL of polymer solution in cell culture medium was added to the cells and incubated for 72 h. A MTT solution (5 mg mL^−1^) was prepared with PBS and filtered with sterile filters (0.2 μm). At the end of the incubation period, 20 μL of MTT solution was added to each well. After incubation (60–75 min), the medium was removed and 200 μL of DMSO was then added to each well to dissolve the formazan crystals. The absorbance was then measured by a microplate reader (LAB Systems Original Multiscan MS) at 570 nm. Cell viability was calculated as the absorbance ratio between treated and untreated control cells. Half-maximal inhibitory concentrations (IC_50_) were calculated using Quest Graph™ IC_50_ Calculator. All experiments were performed in triplicate to determine means and standard deviations (SDs).

### 2.6 *In vivo* evaluations

#### 2.6.1 Ethic protocols

All animal experiments were conducted according to the European rules (86/609/EEC and 2010/63/EU) and the Principles of Laboratory Animal Care and legislation in force in France (Decree No. 2013-118 as of February 1, 2013). Toxicity experiments obtained experimental approval from the Ethical Committee C2EA-26 (Comité d’éthique en expérimentation animale de l’IRCIV, Authorization number APAFIS#7756). *In vivo* efficacy experiments were performed by Oncodesign (Les Ulis, France) as study N°230456. Animal housing and experimental procedures have been conducted according to the French and European Regulations and the National Research Council Guide for the Care and Use of Laboratory Animals. The animal facility is authorized by the French authorities (Dijon: Agreement no. C 21231 011 EA). All animal procedures (including surgery, anesthesia and euthanasia as applicable) used in the current study (230456/ACT1 MIA PaCa-2 SC/Ethical protocol: 2022-03 ONCO SC) have been submitted to the Institutional Animal Care and Use Committee of Oncodesign Services (Oncomet) approved by French authorities [CNREEA agreement no. 91 (Oncodesign Services)].

#### 2.6.2 Local and systemic toxicity

Groups of 3 mice were injected subcutaneously in the interscapular region at day 0 (single dose) or at days 0, 4, 7 and 11 (multiple doses). The different groups are as follows: (i) Gem at 80, 120, 160, 200, 500, 650 and 1000 mg kg^−1^. The doses 80, 120 and 160 mg kg^−1^ were also tested as multiple injections; (ii) polymer prodrug P7 at 60, 500 and 650 mg kg^−1^; (iii) copolymer prodrugs P2 and P3 at 60 mg kg^−1^; (iv) drug-free copolymers P5 and P6 at 15 mg kg^−1^; (v) PBS (negative control). Visual toxicities at the injection site and body weights were monitored daily to follow local and systemic toxicities. After 14 days, mice were euthanized by cervical dislocation and injection sites were withdrawn. Tissue samples were fixed with 4% PFA and embedded in paraffin. Sections (3 μm thick) were deparaffinized and stained with hematoxylin and eosin (H&E), and toluidine blue staining (VWR, France) for the detection of mast cells. For immunofluorescence, slides were incubated overnight at 4 °C with primary antibodies diluted in 0.02% Triton X-100-PBS. Primary antibodies were as follows: rat monoclonal anti-CD3 (Abcam, ab11089; diluted 1 : 100), and mouse anti-Ly6G (BioLegend, France, clone A8; diluted 1 : 50), followed by staining with appropriate secondary antibodies, Alexa FluorTM 488 (Invitrogen, ThermoFisher Scientific A-11008; diluted 1 : 250) and Alexa FluorTM 594 (Invitrogen, ThermoFisher Scientific A-11012; diluted 1 : 250). DNA was visualized upon Hoechst counterstaining (Invitrogen, ThermoFisher Scientific H3570). Slides were scanned using NanoZoomer 2.0-RS digital slide scanner (Hamamatsu, Japan). Images were digitally captured from the scanned slides using NDP.view2 software (Hamamatsu).

#### 2.6.3 Anticancer efficacy

78 healthy female SCID (CB17/lcr-Prkdcscid/lcrlcoCrl) mice, 6-8 weeks old, were obtained from Charles River. Mia PaCa-2 tumor cell implantation was performed 24 to 72 h after a whole-body irradiation with a gamma-source (1.44 Gy (SCID mice, NSG mice), ^60^Co, BioMep, France). Mia PaCa-2 pancreatic tumors were induced by SC injection of 2 × 10^7^ Mia PaCa-2 cells in 200 μL of RPMI 1640 medium into the right flank of mice. At day 17, when tumors reach a mean volume of 100–200 mm^3^, 60 animals out of 78 were randomized into 4 groups of 7–12 animals each. Homogeneity of the mean tumor volume between groups was tested by an analysis of variance (ANOVA). The treatments started on the day of randomization. Treatments were administered either by SC injection in the interscapular region or by IV injection into the caudal vein. A Q7Dx3 treatment schedule was applied (*i.e.*, once a week, 3 consecutive weeks, which corresponds to injections on days 17, 24 and 31)), as follows: (i) Gemzar^IV^ at 60 mg kg^−1^ (Gemzar MTD); (ii) Gem-PAAm (P9^SC^) at 1145 mg kg^−1^ (60 mg kg^−1^ Gem equiv. dose); (iii) Gem-PAAm (P10^SC^) at 2290 mg kg^−1^ (60 mg per kg Gem equiv. dose and (iv) Gem-PAAm (P11^SC^) at 4580 mg kg^−1^ (60 mg per kg Gem equiv. dose). Animal viability and behavior were observed daily, and body weights were measured twice a week. The length and width of the tumor were measured twice a week with calipers. Mice were euthanized by overdosage of gas anesthesia (isoflurane) or CO_2_ induction, followed by cervical dislocation or exsanguination. Necropsy (macroscopic examination) has been performed on all animals euthanized in the study.

#### 2.6.4 Statistics

Statistics were performed using GraphPad Prism (version 8.0.2). Comparison of tumor growth outcomes between groups was analyzed for statistical significance, using two-way ANOVA, with Tukey's *post hoc* test for multiple comparisons.

## 3. Results and discussion

### 3.1 Polymer prodrug synthesis and physicochemical evaluation

The copolymer prodrugs are composed of AAm units, to confer hydrophilicity, and BMDO units, as precursors of ester groups in the copolymer backbone. This copolymerization system was chosen because of the proven UCST properties of P(AAm-*co*-BMDO) copolymers, typically between 23 and 55 °C depending on the BMDO content.^31^ They also exhibit rapid hydrolytic degradation under physiological conditions, faster than that of traditional aliphatic polyesters such as polycaprolactone (PCL), polylactide (PLA) and even poly(lactic-*co*-glycolic acid) (PLGA), which are still regarded as benchmarks in the field of biodegradable polymers. These two properties therefore guarantee: (i) complete water-solubility in SC tissue ensured by a correctly adjusted *T*_cp_ value, a prerequisite for reaching systemic circulation and (ii) efficient excretion of low molar mass copolymer fragments during degradation *in vivo*. Importantly, due to the rapid degradation of these copolymers, we reasoned that triggering water-solubility only after injection in the SC tissue would prevent early degradation of the copolymer during formulation and storage, and hence early release of Gem, which would be caused by increased solvation of the Gem-polymer linker. The rationale on developing UCST polymer prodrug is summarized in Fig. S4.†

#### 3.1.1 Synthesis of Gem-P(AAm-*co*-BMDO) copolymer prodrugs

**Fig. 2:**
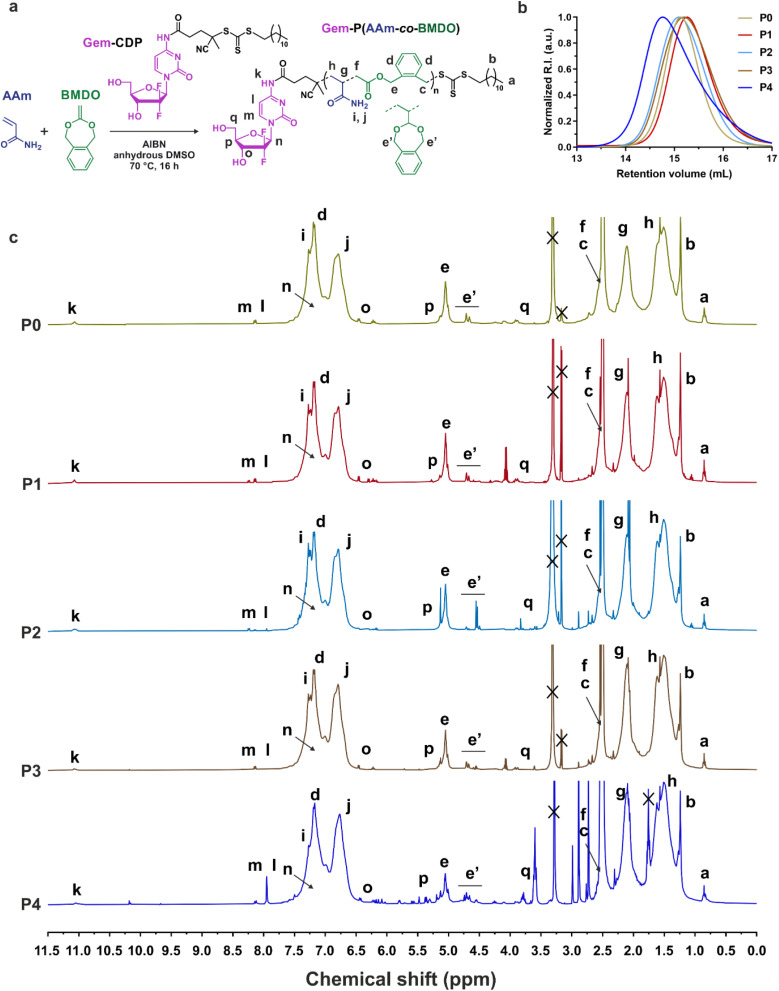
Synthesis and characterization of Gem-P(AAm-*co*-BMDO) copolymer prodrugs. (a) RAFT-mediated copolymerization of AAm and BMDO from Gem-CDP; (b) SEC chromatograms in DMSO of Gem-P(AAm-*co*-BMDO) copolymer prodrugs (P0–P4, Table 1); (c) ^1^H-NMR spectra (300 and 400 MHz, DMSO-*d*_6_) in the 0–11.5 ppm region of Gem-P(AAm-*co*-BMDO) copolymer prodrugs (P0–P4, Table 1).

To install Gem in α-position of the copolymer chain, CDP was conjugated to Gem *via* the formation of an amide bond between the carboxylic group of CDP and the amine group of Gem, using EDC/NHS coupling chemistry. The Gem-CDP RAFT agent was characterized by ^1^H, ^13^C and ^19^F NMR, as well as by HR-MS (see Experimental part), and obtained with an overall yield of 30%. The amide bond was selected as polymer-drug linker to facilitate Gem release *via* protease-driven cleavage,^38^ as these enzymes, such as cathepsin B and D, are often found in abnormally high concentrations in tumors.^39,40^ In addition, its high stability under physiological conditions is expect to prevent early release of Gem, and to enable the safe SC administration of the prodrug.^41^ Gem-P(AAm-*co*-BMDO) copolymer prodrug was obtained by RAFT copolymerization of AAm and BMDO from Gem-CDP in DMSO at 70 °C for 16 h under AIBN initiation (Fig. 2a). Copolymerization was carried out at 0.8 M (total monomer concentration) to avoid a too viscous reaction medium at high monomer conversion,^31^ thus preventing the occurrence of side reactions, which are detrimental to the control of the copolymerization, as well as maximizing ring-opening of BMDO (conversely to a ring-retaining propagation).^42^

A small library of Gem-P(AAm-*co*-BMDO) copolymer prodrugs was obtained by varying the AAm : BMDO molar ratio (from 50 : 50 to 60 : 40) and by targeting an overall number-average degree of polymerization (DP_n_) of 200 (P0–P4, Table 1). The copolymerizations exhibited AAm conversions ranging from 40 to 90%, depending on the BMDO content (the higher *f*_BMDO,0_, the lower the AAm conversion). They were well-controlled, with *M*_n,NMR_ values ranging from 6020 to 9140 g mol^−1^ and fairly low dispersities obtained (*Đ* = 1.3–1.5) (Table 1 and Fig. 2b). ^1^H-NMR and ^19^F-NMR spectra confirmed the presence of Gem on the copolymer structures, due to the presence of specific proton peaks in the 5–11 ppm region (Fig. 2c), as well as a peak characteristic of their two fluorine atoms at 120 ppm (Fig. S5†). Two Gem-free P(AAm-*co*-BMDO) copolymers (P5 and P6, *M*_n,NMR_ = 8840 and 8860 g mol^−1^, *Đ* = 1.2 and 1.1, AAm : BMDO = 56 : 44 and 57 : 43, respectively) were also synthesized as controls by RAFT copolymerization of AAm and BMDO from CDP, under the same experimental conditions as for the synthesis of Gem-P(AAm-*co*-BMDO) (Fig. S6† and Table 1).

**Table 1:** Macromolecular characteristics of Gem-P(AAm-*co*-BMDO) and Gem-PAAm (co)polymer prodrugs and their drug-free counterparts P(AAm-*co*-BMDO) and PAAm, synthesized by RAFT-mediated polymerization

Entry	Initial monomer feed, *f*_0_ (mol%)		Copolymer composition, *F*^a^ (mol%)		Open BMDO^b^ (mol%)	AAm conversion^c^ (mol%)	*M*_n,NMR_ d (g mol^−1^)	*M*_n,SEC_ e (g mol^−1^)	*Đ* e	Drug loading^a^ (wt%)	*T*_cp_ g from UV (°C)		*T*_cp_ i from DLS (°C)
	AAm	BMDO	AAm	BMDO							Heating	Cooling	
P0	50	50	84.6	15.4	90	40	7300	10 000	1.20	4.1	64	62	—j
P1	55	45	90.2	9.8	91	70	6020	8310	1.25	5.0	55	56	41
P2	56	44	90.6	9.4	90	87	9000	10 300	1.26	3.3	37	27	31
P3	57	43	92.2	7.8	88	90	8300	9100	1.32	3.6	22	22	26
P4	60	40	92.7	7.3	89	61	9140	10 880	1.52	3.3	8	8	6
P5	56	44	90.7	9.3	91	80	8840	10 010	1.20	—f	40	34	—j
P6	57	43	92.1	7.9	90	88	8860	10 890	1.14	—f	18	18	—j
P7	100	0	100	0	n.a.	79	9700	17 250	1.52	3.1	—h	—h	—h
P8	100	0	100	0	n.a.	97	8930	16 210	1.24	—f	—h	—h	—h
P9	100	0	100	0	n.a.	97	7700	6180	1.34	3.9	—h	—h	—h
P10	100	0	100	0	n.a.	94	12 410	12 260	1.26	2.4	—h	—h	—h
P11	100	0	100	0	n.a.	91	27 490	21 340	1.29	1.1	—h	—h	—h

a Determined by ^1^H-NMR after purification, according to: MW Gem/(MW Gem + *M*_n,NMR_ polymer prodrug).

b Determined by ^1^H-NMR after precipitation by integrating the 2H (–NH_2_) of AAm, the 4H (aromatic protons) of open and closed BMDO (6.5–7.5 ppm), the 2H of open BMDO (4.9–5.2 ppm) and the 4H of closed BMDO (4.5–4.8 ppm). n.a. = not applicable.

c Determined by ^1^H-NMR (300 MHz) by integrating the 2H of AAm (6.02–6.24 ppm) at *t* = 0 and 16 h.

d Determined by ^1^H NMR after purification by integrating the 3H from the CH_3_ moiety of the RAFT agent C_12_ alkyl chain (0.86 ppm), the 1H of AAm (2.1 ppm), the 2H of open BMDO (4.9–5.2 ppm) and the 4H of closed BMDO (4.5–4.8 ppm). Note that this method is only accurate for high living chain fractions.

e Determined by SEC in DMSO.

f Copolymers obtained from the Gem-free CDP RAFT agent.

g Determined from the maximum of the first derivative of the heating and cooling curves obtained by UV-Vis spectroscopy at 1 °C min^−1^ and at 10 mg mL^−1^ in MilliQ water.

h No thermosensitivity in absence of BMDO units.

i Determined by DLS from the inflection point of the *D*_z_*vs.* temperature curve upon cooling at 10 mg mL^−1^ in MilliQ water.

j Not determined.

The composition of the different purified copolymers as well as the average percentage of ring-opened and closed BMDO units were determined by ^1^H-NMR spectroscopy. All copolymer prodrugs (P0–P4, Fig. 2c and Table 1) and drug-free copolymers (P5–P6, Fig. S6† and Table 1) exhibited very high molar fractions of ring-opened BMDO (88–91 mol%), suggesting a great susceptibility to hydrolysis. When the initial molar fraction of BMDO (*f*_BMDO,0_) is varied from 0.4 to 0.5, the BMDO contents in the copolymers (*F*_BMDO_) was in the 0.07–0.15 range, as expected from the unfavorable reactivity ratios (*r*_BMDO_ = 0.23 and *r*_AAm_ = 13.02)^31^ (Table 1).

#### 3.1.2 Degradation of Gem-P(AAm-*co*-BMDO) prodrugs

**Fig. 3:**
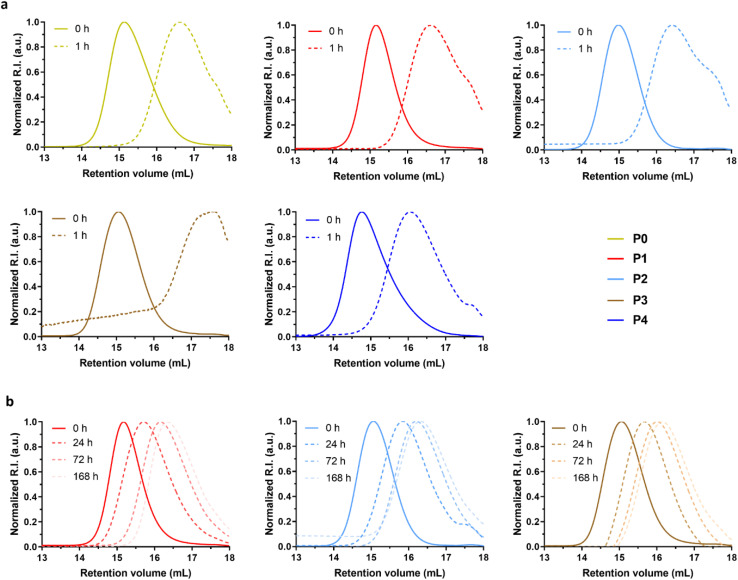
Degradation studies of Gem-P(AAm-*co*-BMDO) copolymer prodrug. (a) Evolution of the SEC chromatograms at *t* = 0 h (solid line) and *t* = 1 h (dotted line) during hydrolytic degradation under accelerated conditions (aqueous KOH 5 wt% solution, room temperature) of Gem-P(AAm-*co*-BMDO) copolymer prodrugs P0–P4 (Table 1); (b) evolution of the SEC chromatograms at different times (0, 24, 72 and 168 h) during hydrolytic degradation under physiological conditions (PBS, pH 7.4, 37 °C) of Gem-P(AAm-*co*-BMDO) prodrug P1–P3 (Table 1).

Degradation of Gem-P(AAm-*co*-BMDO) copolymer prodrugs P0–P4 was first carried out under accelerated hydrolytic conditions (aqueous KOH 5 wt% solution) to confirm the presence of ester groups in the main chain. After degradation for 1 h at room temperature, clear shifts of the SEC traces towards lower molar mass values were obtained (Fig. 3a), which corresponds to decreases in *M*_n_ of 87–94%, close to the expected values (Table 2). Importantly, as expected from the rapid hydrolytic degradation of P(AAm-*co*-BMDO) copolymers under physiological conditions,^31^ Gem-P(AAm-*co*-BMDO) copolymer prodrugs also exhibited significant degradation in PBS at 37 °C and pH 7.4 after only 7 days (Fig. 3b), illustrated by decrease in *M*_n_ of 77–82%, which were also very close to the theoretical values (Table 2). These results therefore confirmed the significant insertion of ester groups in the copolymer prodrug backbone, the rapid copolymer degradation under physiological conditions as well as the absence of detrimental effect of Gem on the degradation. The average number of consecutive AAm units at low theoretical monomer conversion was determined to vary between ∼14 and ∼21 for P0–P4, respectively (Table S1†), showing that, despite the gradient nature of the copolymerization, oligomers with very low *M*_n_ are obtained in the worst case after degradation.

**Table 2:** Macromolecular characteristics of Gem-P(AAm-*co*-BMDO) copolymer prodrugs before and after hydrolytic degradation under accelerated and physiological conditions

Entry	*M*_n,SEC_ a (g mol^−1^)	*Đ* a	*M*_n,deg. accel._ b (g mol^−1^) (% *M*_n_ loss)	*Đ*_deg accel._ b	*M*_n,deg. physio._ c (g mol^−1^) (% *M*_n_ loss)	*Đ*_deg. physio._ c	*M*_n,deg.theo._ d (g mol^−1^) (% *M*_n_ loss)
P0	10 000	1.20	600 (−94%)	1.78	—e	—e	1400 (−86%)
P1	8310	1.25	1050 (−87%)	2.04	1460 (−82%)	1.82	870 (−90%)
P2	10 300	1.26	1 140 (−89%)	2.17	1700 (−83%)	1.94	970 (−91%)
P3	9100	1.32	860 (−91%)	2.27	2080 (−77%)	1.83	1100 (−88%)
P4	10 880	1.52	1000 (−91%)	2.02	—e	—e	2200 (−80%)

a Determined by SEC in DMSO.

b Determined by SEC in DMSO after hydrolytic degradation in aqueous KOH 5 wt% solution for 1 h.

c Determined by SEC in DMSO after degradation under physiological conditions (PBS, 37 °C, 7 days).

d Determined according to: *M*_n_,_deg. theo._ = ([1/(open BMDO × *F*_BMDO_)] − 1) × MW_(AAm)_ + MW_(BMDO)_, with MW being the molecular weight of the considered monomers.

e The degradation was not carried out.

#### 3.1.3 UCST properties of Gem-P(AAm-*co*-BMDO) copolymer prodrugs

The thermoresponsive behavior of the Gem-based copolymer prodrugs was then studied to: (i) evaluate the influence of the presence of the Gem moiety on the UCST properties of the copolymer prodrugs when compared to those of the drug-free P(AAm-*co*-BMDO) copolymers^31^ and (ii) find the best AAm/BMDO molar ratio to induce a lower *T*_cp_ than the SC tissue temperature (33–35 °C), to ensure complete solubility after SC administration as required for reaching systemic circulation. The *T*_cp_ values were determined at 10 mg mL^−1^ in water by measuring the absorbance of the copolymer solution over time by UV-Vis spectroscopy (called UV transmittance), followed by the calculation of its first derivative as a function of the temperature, and also by monitoring the *D*_z_ value of the copolymer solution over time by DLS at different temperatures.

**Fig. 4:**
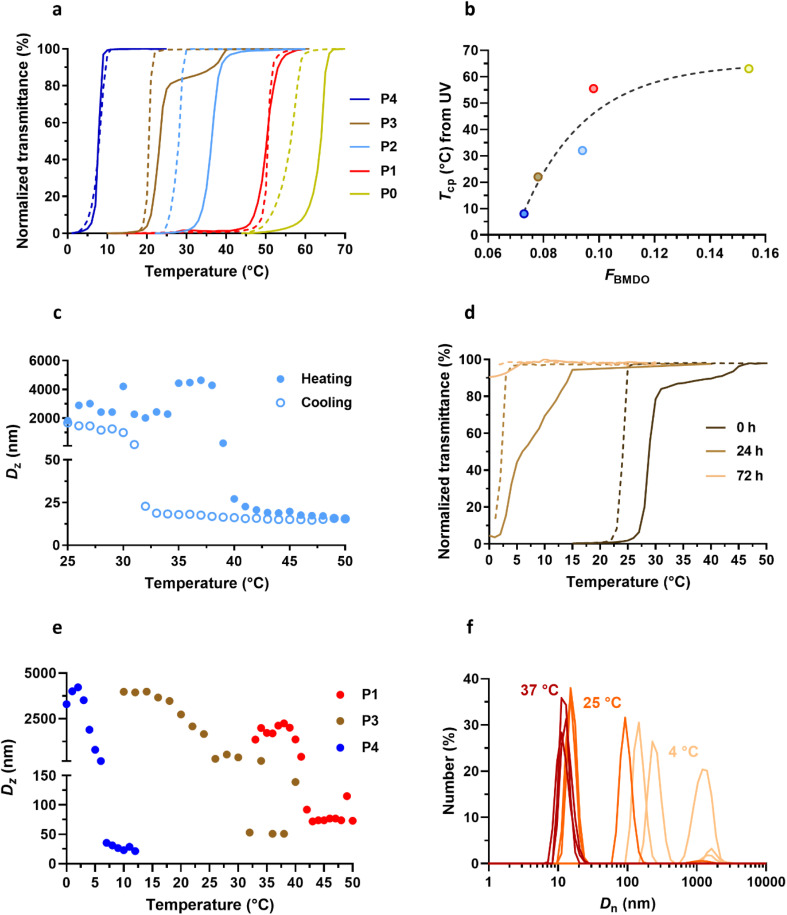
Thermosensitive properties of Gem-P(AAm-*co*-BMDO) copolymer prodrugs. (a) Variation of the UV transmittance in MilliQ water as function of the temperature of Gem-P(AAm-*co*-BMDO) copolymer prodrugs P0–P4 solutions at 10 mg mL^−1^ upon heating (solid lines) and cooling (dotted lines); (b) evolution of *T*_cp_ measured by UV (average between heating and cooling values, see Table 1) as a function of *F*_BMDO_ for copolymers P0–P4; (c) DLS evolution of the intensity-weighted mean diameter (*D*_z_) with temperature of Gem-P(AAm-*co*-BMDO) copolymer prodrug P2 at 10 mg mL^−1^ in MilliQ water upon cooling (empty dots) and heating (plain dots); (d) variation of the UV transmittance with temperature of Gem-P(AAm-*co*-BMDO) copolymer prodrug P3 solution upon heating (solid lines) and cooling (dotted lines) at 10 mg mL^−1^ in MilliQ water at *t* = 0, 24 and 72 h; (e) DLS evolution of *D*_z_ with temperature of Gem-P(AAm-*co*-BMDO) copolymer prodrugs P1, P3 and P4 at 10 mg mL^−1^ in MilliQ water upon cooling; (f) evolution of the number-weighted mean diameter (*D*_n_) (*n* = 3) of Gem-P(AAm-*co*-BMDO) copolymer prodrug P3 at 10 mg mL^−1^ in MilliQ water at *T* = 4 °C (beige), 25 °C ∼ *T*_cp_ (orange) and 37 °C (red).

Overall, Gem-P(AAm-*co*-BMDO) prodrugs P0–P4 exhibited sharp UCST transitions upon cooling and heating in water in the 8–64 °C range depending on *F*_BMDO_ (Fig. 4a), confirming that the BMDO contents investigated (*F*_BMDO_ = 0.073–0.154) were sufficient to confer thermosensitivity. The transmittance curves of P0–P4 shifted towards higher *T*_cp_ values as function of *F*_BMDO_ (Fig. 4a), following an exponential plateau curve and suggesting some predictability of the *T*_cp_ value when varying the copolymer composition (Fig. 4b). These values were around 10 to 20 °C higher than those obtained in the absence of Gem for similar BMDO contents.^31^ It is also worth noting the sensitivity of the system, since a wide range of *T*_cp_ values was obtained by reducing the BMDO content from 15.4% to just 7.3% (Fig. 4b and Table 1). Interestingly, the copolymer prodrug P2 (*F*_BMDO_ = 0.094, Table 1) has a *T*_cp_ value that spans the temperature of the SC tissue (*T*_cp_ = 27 and 37 °C upon cooling and heating, respectively), with hysteresis between heating and cooling cycles similar to that observed by DLS (Fig. 4a and c). This hysteresis could be attributed to the onset of polymer degradation during measurement, altering the balance between polymer–polymer and polymer–water interactions. To better understand the evolution of *T*_cp_ over time, the *T*_cp_ of P3 in water was followed by UV transmittance measurement for 3 days (Fig. 4d). *T*_cp_ shifted from 27 to 5 °C after 24 h and the UCST properties disappeared completely after 72 h, confirming the influence of polymer degradation on thermosensitivity.

Measurement of *D*_z_ by DLS of P1–P4 at different temperatures gave *T*_cp_ values in agreement with those determined by UV transmittance measurements (Table 1 and Fig. 4c and e). In addition, the evolution of *D*_n_ with temperature confirmed the solubility of copolymer prodrugs above *T*_cp_, as shown by the presence of 10 nm-unimers, while much larger objects were measured at lower temperatures (*T* ≪ *T*_cp_) (Fig. 4f). Note also the coexistence of 15 nm-unimers and 100 nm-nano-objects at *T* = *T*_cp_ for P3, suggesting partial (or ongoing) solubilization of the copolymer prodrug.

### 3.2 *In vitro* characterization

The *in vitro* biological evaluation (*i.e.*, release, cytotoxicity and injectability) was performed using P2 and P3 polymer prodrugs as they exhibit both lower *T*_cp_ values than that of the SC tissue (*T*_cp,DLS_ = 31 and 26 °C, respectively) and the highest ester contents, making them promising candidates for future *in vivo* applications. This study has been completed by the evaluation of non-degradable (*i.e.*, BMDO-free) polymer prodrug Gem-PAAm P7 and its drug-free counterpart PAAm P8.

#### 3.2.1 Drug release

**Fig. 5:**
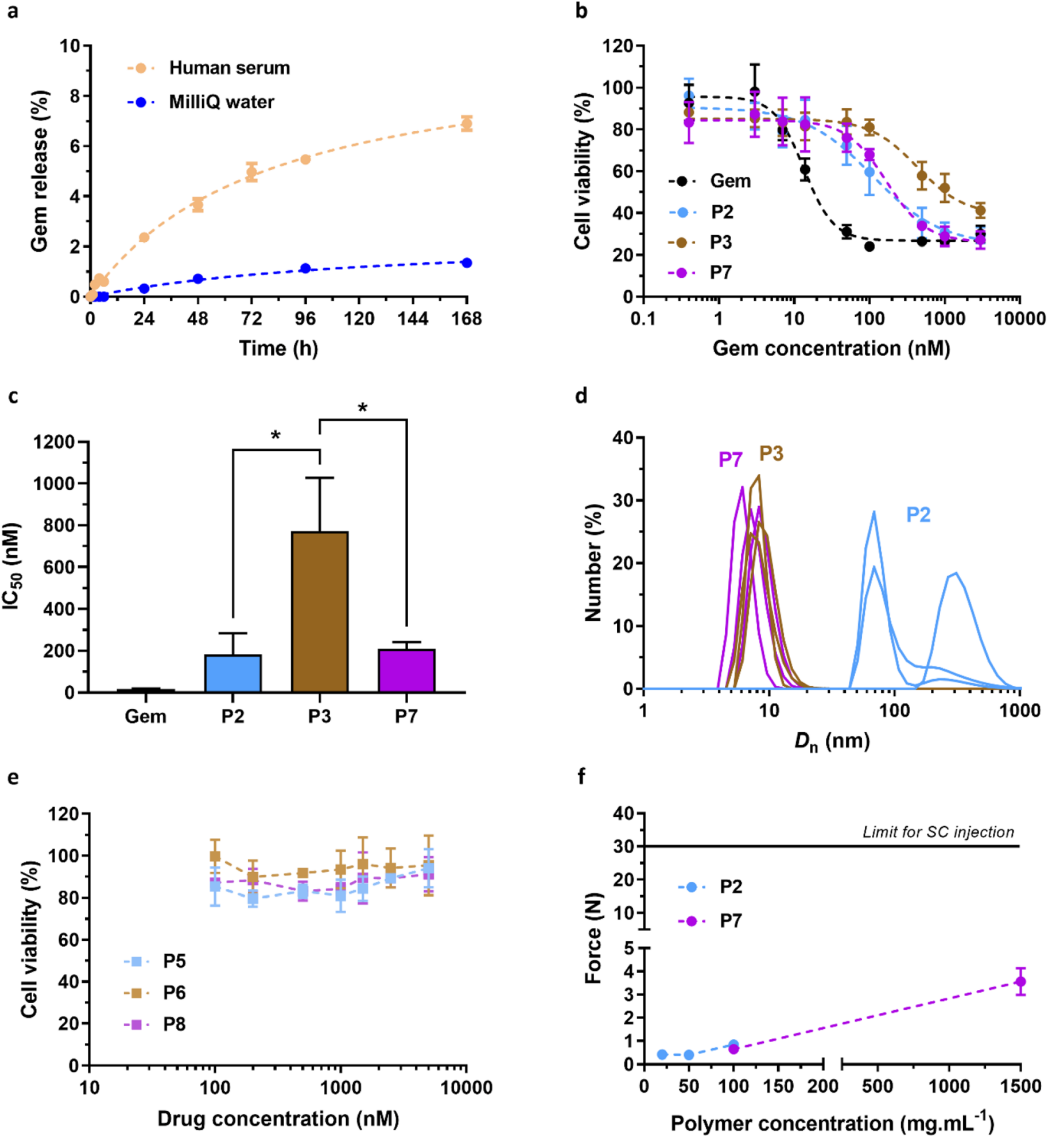
*In vitro* biological evaluation of Gem-P(AAm-*co*-BMDO), Gem-PAAm, P(AAm-*co*-BMDO) and PAAm. (a) HPLC release profiles of Gem from Gem-P(AAm-*co*-BMDO) P2 determined at 37 °C in MilliQ water (blue) and in human serum (orange); (b) cell viability (MTT test) of Mia PaCa-2 cells after incubation for 72 h with increasing concentrations of Gem, Gem-P(AAm-*co*-BMDO) P2 and P3 and Gem-PAAm P7; (c) determination of the IC_50_ for the four tested conditions (unpaired two-tailed *t* test; *p < 0.05, P2, P7*vs.*P3); (d) number-weighted mean diameter (*D*_n_) of aqueous solutions of P2, P3 and P7 at 1.23 mg mL^−1^ stored at 37 °C; (e) cell viability (MTT test) of Mia PaCa-2 cells after incubation for 72 h with increasing concentrations of P(AAm-*co*-BMDO) P5 and P6, and PAAm P8; (f) force needed using a syringe fitted with a 26 G needle to inject an aqueous solution of P2 and P7 at different concentrations (20–100 mg mL^−1^ for P2 and 100–1500 mg mL^−1^ for P7). The horizontal dashed line of 30 N is considered as the maximum acceptable injection force for SC administration.^46^ The values are expressed as the means ± SD.

Gem release from Gem-P(AAm-*co*-BMDO) copolymer prodrug P2 was monitored at 37 °C in MilliQ water and human serum to evaluate the influence of hydrolytic and enzymatic cleavages, respectively, on drug release kinetics. A slow release was obtained in MilliQ water (1.4% after 1 week), whereas human serum significantly accelerated the Gem release, which progressively reached 7% after 1 week (Fig. 5a). Faster Gem release in human serum is likely assigned to the presence of specific enzymes able to cleave the amide bond which served as linker between Gem and the copolymer. Moreover, it could also be related to the concomitant hydrolytic degradation of ester bonds from BMDO units, facilitating enzyme access to the amide function. It is important to note that such a slow and prolonged release of Gem should be beneficial for SC administration, as it would avoid an early release of the drug into the SC tissue, thus avoiding cutaneous toxicity.

#### 3.2.2 Cytotoxicity of Gem-P(AAm-*co*-BMDO) prodrugs

Cell viability experiments (MTT assays) were performed to assess the cytotoxicity of Gem-P(AAm-*co*-BMDO) copolymer prodrugs P2 and P3 on pancreatic cancer cells Mia PaCa-2, as a relevant clinical model for Gem.^43^P2 and P3 differ from their *T*_cp_ value (∼32 °C *vs.* 22 °C, respectively, Table 1) and were tested to assess the influence of polymer solubility on cytotoxicity, as P3 is completely soluble at the temperature of the MTT assay, conversely to P2. Prior to the MTT assay, we confirmed that the thermosensitivity of P2 and P3 (*T*_cp_ of ∼32 °C and 22 °C, respectively) and the drug-free counterparts P5 and P6 (*T*_cp_ of 30 °C and 11 °C, respectively) was well preserved at the concentration of the mother solution (1.23 mg mL^−1^) used for MTT assay, as for 10 mg mL^−1^ (Fig. S7† and Table 1).

Both P2 and P3 led to significant cytotoxicity, with IC_50_ values of 180 nM and 770 nM, respectively (Fig. 5b and c). As is often the case with polymer prodrugs, P2 and P3 exhibited higher IC_50_ values than that of free Gem (IC_50_ = 18 nM), due to the cleavage step of the Gem-polymer covalent linkage to release the parent drug. Importantly, P3 was significantly less cytotoxic than P2, with an IC_50_ value 4-times higher than that of P2 (Fig. 5c). As expected from their different *T*_cp_ values, DLS evaluation of P2 and P3 in solution at 37 °C showed different colloidal properties, as P2 was characterized by coexistence of nano-objects of different sizes (*D*_n_ = 190 nm and 340 nm), whereas P3 gave unimers of ∼10 nm in size (Fig. 5d). This can have an impact on cellular uptake mechanisms and eventually on cytotoxicity, as nano-objects have been shown to follow different endocytosis pathways depending on their size^44^ and Mia PaCa-2 are associated with enhanced macropinocytosis (*i.e.*, which favors internalization of nano-objects >250 nm in size).^45^ Moreover, fully soluble Gem-P(AAm-*co*-BMDO) unimers P3 are likely to be much prone to degradation throughout the MTT assay, producing small oligomer prodrugs, compared to Gem-P(AAm-*co*-BMDO) aggregates P2. We also showed that Gem-free copolymers P(AAm-*co*-BMDO) P5 and P6 were not cytotoxic up to at least 5 μM, ruling out potential cytotoxicity from the copolymer itself (Fig. 5e).

To study independently the influence of degradation on cytotoxicity, we also synthesized and evaluated two non-degradable, BMDO-free copolymers of similar chain length: a Gem-PAAm polymer prodrug P7 (*M*_n,NMR_ = 9700 g mol^−1^, *Đ* = 1.52) and its Gem-free counterpart PAAm P8 (*M*_n,NMR_ = 8930 g mol^−1^, *Đ* = 1.24) (Fig. S8† and Table 1). As expected, due to the absence of BMDO units in the polymer chains, P7 and P8 did not exhibit UCST behavior. Whereas PAAm P8 was also not cytotoxic up to at least 5 μM (Fig. 5e), Gem-PAAm P7 produced ∼10 nm-unimers (Fig. 5d), but exhibited the same IC_50_ value as Gem-P(AAm-*co*-BMDO) P2 aggregates and thus remained more cytotoxic than Gem-P(AAm-*co*-BMDO) P3 unimers (Fig. 5b and c). This result could be attributed to the persistence of the PAAm chain in Gem-PAAm P7 compared to the rapid degradation of Gem-P(AAm-*co*-BMDO) P3 into small oligomers.

#### 3.2.3 Injectability

Prior to *in vivo* assessment, the injectability (*i.e.*, force required for injection) of the polymer prodrugs was evaluated to ensure they can be injected subcutaneously under standard conditions. This physicochemical parameter remains very important to study for SC injection as only small volumes (up to ∼2 mL) are tolerated, which requires the administration of sufficiently concentrated solutions to obtain a therapeutic effect.

The injectability of aqueous solutions of Gem-P(AAm-*co*-BMDO) P2 and Gem-PAAm P7 was measured as function of the concentration with a 26 G × 1/2′′ needle, which is within the size range suitable for humans (25–27 G). Previous work demonstrated that the force needed to inject PAAm (*M*_n_ = 37 000 g mol^−1^) remained well below the maximum tolerated force of 30 N (<5 N at 200 mg mL^−1^), whereas injection of Ptx-PAAm considerably increased the required force (∼20 N at 100 mg mL^−1^), which even exceeded the 30 N limit at 200 mg mL^−1^.^20^ This was assigned to intermolecular hydrophobic interactions between the Ptx moieties and/or between the Ptx moieties and the C_12_ alkyl chain from the RAFT end groups. Herein, P2 and P7 were designed with a hydrophilic drug and a lower *M*_n_ (∼10 000 g mol^−1^), to maintain low viscosity and allow high doses to be administered subcutaneously. At a concentration up to 100 mg mL^−1^, which corresponds to a dose in Gem of ∼50 mg kg^−1^, the SC injection of both polymer prodrugs required a very low force of ∼1 N (Fig. 5f), which is comparable to the force required for PAAm alone.^20^ Not only this result confirms the absence of hydrophobic interactions between the Gem groups and/or between the Gem groups and the terminal C_12_ alkyl chains, but it also ruled out detrimental effect of hydrophobic BMDO units on the viscosity of P2. The Gem-based polymer prodrugs synthesized in this work can therefore be injected more easily *via* the subcutaneous route than the Ptx-PAAm prodrugs. To assess injectability at very high concentrations, P7 was injected at 1500 mg mL^−1^ (corresponding to a Gem equivalent dose of ∼650 mg kg^−1^), which led to a 3-fold increase of the force value (3.6 N), but remained well below the limit of 30 N. Therefore, high doses of water-soluble, Gem-based polymer prodrugs can be easily administered subcutaneously.

### 3.3 *In vivo* evaluations

#### 3.3.1 Systemic toxicity

The systemic toxicity of free Gem and polymer prodrugs P2, P3 and P7 was evaluated in mice to determine the MTD, defined as the highest dose that does not produce unacceptable toxicity, followed by examination of the acute local toxicity at the injection site.

**Fig. 6:**
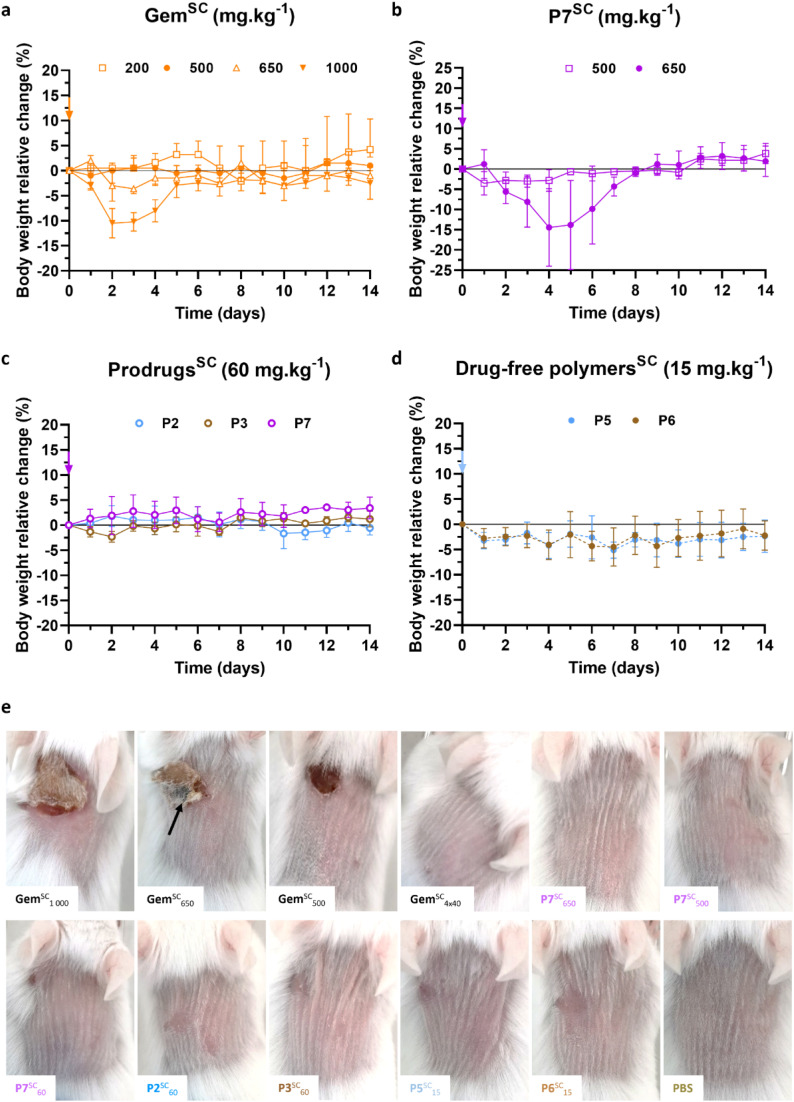
SC injection of Gem, Gem-based polymer prodrugs and control polymers to mice. Relative body weight changes of mice as a function of time after single SC injection of: (a) free Gem at different doses (200, 500, 650 and 1000 mg kg^−1^); (b) Gem-PAAm P7 at 500 and 650 mg kg^−1^; (c) Gem-P(AAm-*co*-BMDO) P2, P3 and Gem-PAAm P7 at 60 mg kg^−1^, and (d) drug-free P(AAm-*co*-BMDO) P5 and P6 at 15 mg kg^−1^ (equiv. Gem; that is 85 mg mL^−1^ in terms of polymer concentration). The values are expressed as the means ± SD (*n* = 3); (e) representative pictures of mice 8 days after SC injection of Gem at 1000, 650, 500 mg kg^−1^ (single injection) and 160 mg kg^−1^ (4 injections), Gem-PAAm P7 at 650, 500 and 60 mg kg^−1^ (single injection), Gem-P(AAm-*co*-BMDO) P2 and P3 at 60 et 15 mg kg^−1^ (single injection), drug-free P(AAm-*co*-BMDO) P5 and P6 at 15 mg per kg equiv. Gem (single injection), and PBS (single injection). The black arrow indicates necrosis zone.

As the MTD of Gem injected intravenously (Gem^IV^) is about 80 mg kg^−1^ after four IV injections,^47^ increasing concentrations of Gem from 80 to 160 mg kg^−1^ were injected subcutaneously (Gem^SC^) to healthy mice following two different injection protocols: (i) a single injection at day 0, or (ii) four injections of a quarter dose each at days 0, 4, 7 and 11. Notably, such doses of Gem^SC^ did not lead to body weight loss, either using one unique or four injections (Fig. S9a–c†). Thus, higher doses of Gem^SC^ from 200 to 1000 mg kg^−1^ were injected using the most convenient single injection protocol, as no difference was observed between the two protocols. While doses from 200 to 650 mg kg^−1^ were well tolerated, a significant body weight loss was observed at 1000 mg kg^−1^ (Fig. 6a), indicating a MTD for Gem^SC^ ranging between 650 and 1000 mg kg^−1^. These results showed that the SC route achieved higher MTD values than the IV route, probably due to a reduction in the *C*_max_ value (defined as the highest concentration of a drug), caused by the time required for the Gem to diffuse from the SC tissue into the systemic circulation.

Gem-PAAm P7 was then injected subcutaneously (P7^SC^) at 500 and 650 mg kg^−1^ to healthy mice *via* a single injection (Fig. 6b) to determine its MTD. P7^SC^ did not induce significant body weight loss (<5%) at 500 mg kg^−1^, which can already be considered as a very high drug concentration for SC administration. However, a transient decrease in body weight of 14% (which returned to normal after 3 days for all mice) was observed at 650 mg kg^−1^ after two days, suggesting similar MTD than Gem^SC^.

Gem-P(AAm-*co*-BMDO) P2 and P3, and Gem-PAAm P7 were then SC-injected at 60 mg kg^−1^ (Fig. 6c), while drug-free P(AAm-*co*-BMDO) copolymers P5 and P6 were SC-injected at 15 mg per kg equiv. Gem (that is 85 mg mL^−1^ in terms of polymer concentration) (Fig. 6d). This injection protocol was carried out to evaluate their toxicity at doses comparable to clinical use of Gem^IV^, known under the brand name Gemzar® (Gemzar) from Lilly France SA,^48^ that is 1000 mg.m^−2^, which is equivalent to 20 mg kg^−1^ for standard human weight and body surface. Importantly, none of these prodrugs and drug-free copolymers led to significant body weight loss compared to untreated mice which received PBS (Fig. S9d†).

#### 3.3.2 Acute local toxicity

**Fig. 7:**
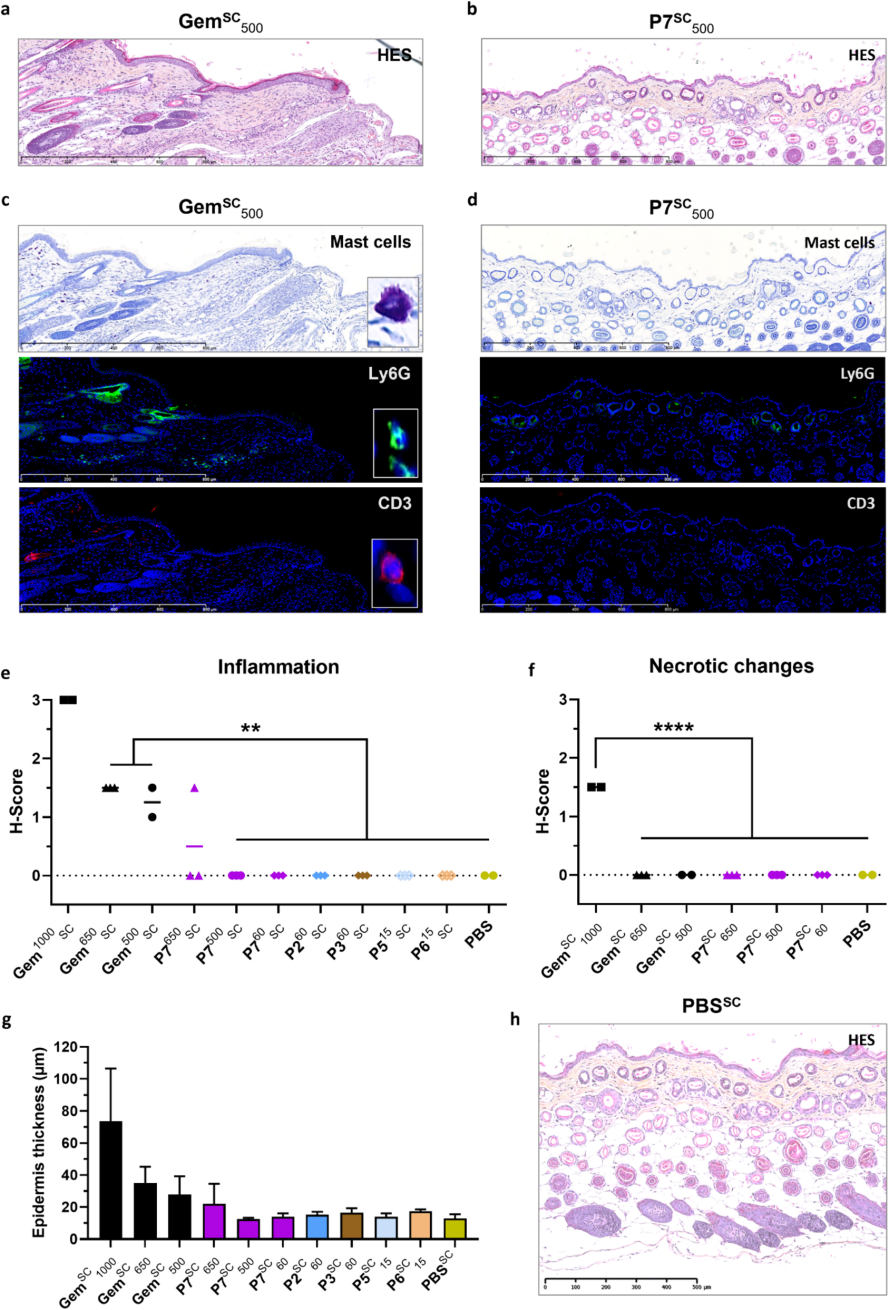
Histological evaluation of the injection site after SC administration of Gem, Gem-PAAm, Gem-P(AAm-*co*-BMDO) and PBS to mice. Representative HES-stained sections of skin sections after 14 days of mice at the injection site after SC administration of: (a) Gem and (b) Gem-PAAm P7 at 500 mg kg^−1^. Representative immunofluorescence images of skin sections after 14 days of mice at the injection site after SC administration of: (c) Gem and (d) Gem-PAAm P7 at 500 mg kg^−1^, using primary antibodies anti-CD3 and anti-Ly6G, and toluidine blue staining for the detection of mast cells respectively. (e) Histopathological scoring (H-Score) of tissular inflammation in mice after SC injection of Gem at 1 000, 650 and 500 mg kg^−1^, Gem-PAAm P7 at 650, 500 and 60 mg kg^−1^, Gem-P(AAm-*co*-BMDO) P2 and P3 at 60 mg kg^−1^, P(AAm-*co*-BMDO) P5 and P6 at 15 mg kg^−1^ and PBS. The values are expressed as the means ± SD (*n* = 3). Unpaired two-tailed *t*-test; *p < 0.01 (Table S2†). (f) Histopathological scoring (H-Score) of necrotic changes in mice after SC injection of Gem at 1000, 650 and 500 mg kg^−1^, Gem-PAAm P7 at 650, 500 and 60 mg kg^−1^ and PBS. The values are expressed as the means ± SD (*n* = 3). Unpaired two-tailed *t*-test; ****p < 0.0001 (Table S2†). (g) Measurement of the epidermis' thickness from HES-stained sections of skin sections after 14 days after SC administration of Gem at 1 000, 650 and 500 mg kg^−1^, Gem-PAAm P7 at 650, 500 and 60 mg kg^−1^, Gem-P(AAm-*co*-BMDO) P2 and P3 at 60 mg kg^−1^, P(AAm-*co*-BMDO) P5 and P6 at 15 mg kg^−1^ and PBS. The values are expressed as the means ± SD after 10 measurements per sample (*n* = 3). (h) Representative HES-stained sections of skin sections after 14 days of mice at the injection site after SC administration of PBS.

The local toxicity at and near the injection site was first evaluated after SC administration of free Gem and Gem-PAAm P7 at high doses. Gem^SC^ at 500, 650 and 1000 mg kg^−1^ induced severe local inflammation, few granulomatous foci in the dermis, some necrotic and/or dark areas and alopecia (*i.e.*, absence of hair follicle) that persisted for at least 2 weeks, as shown by representative skin pictures at days 1, 8 and 14 (Fig. 6e and S10†). Histopathological examination of HES-stained sections of skin samples 14 days after injection (Fig. 7a and S11†) confirmed the local inflammation (Fig. 7e and Table S2†), some necrotic areas for the highest dose in Gem^SC^ (Fig. 7f and Table S2†), and demonstrated large increase in the epidermis mean thickness (*i.e.*, hyperplasia) (Fig. 7g), compared to normal thickness of mice injected with PBS (Fig. 7h). Immunofluorescence of skin sections after SC injection of Gem^SC^ at 500 mg kg^−1^ revealed an important inflammation characterized by the abnormally high presence of mastocytes, neutrophils and lymphocytes T (Fig. 7c).

Remarkably, Gem-PAAm P7^SC^ did not induce local toxicity at or near the injection site at the highest doses tested of 500 and 650 mg kg^−1^, as shown on representative pictures at days 1, 8 and 14 (Fig. 6e and S10†). Moreover, HES-stained sections of skin samples after 14 days did not show significant histopathological lesion or hyperplasia at 500 mg kg^−1^ (Fig. 7b and e–g), while one mouse out of 3 treated by Gem-PAAm P7^SC^ at the MTD (650 mg kg^−1^) exhibited moderate inflammation. Immunofluorescence of skin sections after SC injection of Gem-PAAm P7^SC^ at 500 mg kg^−1^ confirmed the absence of inflammation (Fig. 7d). Taken together, these promising results pave the way for safe SC administration of Gem at high doses under the form of water-soluble polymer prodrugs, since the cutaneous toxicity of free Gem has been suppressed.

Lower, more clinically relevant doses of Gem-P(AAm-*co*-BMDO) P2, P3 and Gem-PAAm P7 (60 mg kg^−1^), and drug-free counterparts P(AAm-*co*-BMDO) P5 and P6 (15 mg kg^−1^) did not induce any local toxicity at and near the injection site, as illustrated by pictures at days 1, 8 and 14 (Fig. 6e and S10†). Slight transient skin irritations appeared in isolated cases but did not persist after a few days. HES-stained sections of skin samples after 14 days (Fig. S11†) showed that the integrity of the SC tissue was preserved and that there was no inflammation (Fig. 7e) or necrosis (Fig. 7f), with the exception of a very small epidermal hyperplasia (Fig. 7g). This study also highlighted the harmlessness of adding BMDO to the polymer backbone, as no difference in toxicity was observed with or without BMDO in the copolymer (see P2 and P3*vs.*P7).

#### 3.3.3 Anticancer efficacy

Due to the promising results of the evaluation of the systemic and local toxicity after SC administration of P2, P3 and P7, Gem-PAAm prodrugs were selected for anticancer efficacy studies, as a good model for Gem-P(AAm-*co*-BMDO) prodrugs and for its ease of synthesis on a gram scale. A small library of Gem-PAAm prodrugs of different chain lengths (P9: *M*_n,NMR_ = 7700 g mol^−1^, *Đ* = 1.34; P10: 12 410 g mol^−1^, *Đ* = 1.26 and P11: 27 490 g mol^−1^, *Đ* = 1.29, see Fig. S12† and Table 1) was synthesized on a gram scale and high yield (58–84%), demonstrating the robustness of the synthesis protocol for obtaining large quantities. Chain length variation was intended to investigate the influence of polymer chain length on anticancer efficacy, as this parameter it is expected to have an impact on biodistribution, body excretion and interaction with the immune system.^49^ Importantly, all molar masses were chosen to be below the kidney filtration threshold (∼6 nm in size, which correspond to ∼40 kg mol^−1^ for PEG^50–52^), to facilitate renal excretion. Varying the chain length also gave the possibility to target different drug loadings (3.9, 2.4 and 1.1 wt% for P9–P11, respectively).

**Fig. 8:**
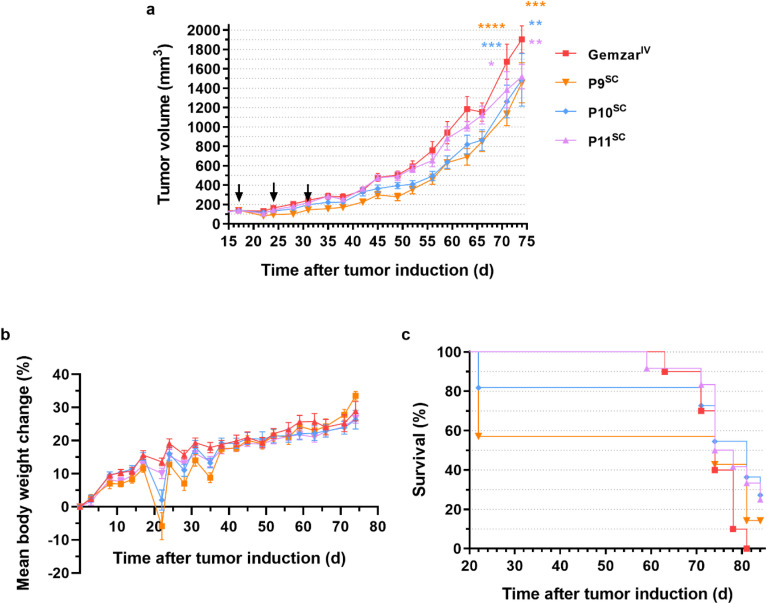
Evaluation of the anticancer efficacy of SC-injected water-soluble Gem-based polymer prodrugs and IV-injected Gemzar®. (a) Tumor growth evolution with time after tumor induction [the values are expressed as the means ± SEM (*n* = 7–12 per group). Two-way ANOVA, with Tukey's correction for multiple comparisons between Gemzar^IV^ and prodrug groups at days 71 and 74; *p ≤ 0.05, **p ≤ 0.01, ***p ≤ 0.002, ****p < 0.0001]; (b) body weight evolution with time after tumor induction [the values are expressed as the means ± SEM (*n* = 7–12 per group)] and (c) survival rate evolution with time after tumor induction [the values are expressed as the means ± SEM] of mice bearing Mia Pa-Ca 2 xenografts after injection of Gemzar^IV^ at 60 mg kg^−1^, and injection of P9^SC^–P11^SC^ at 60 mg per kg Gem equiv. dose, on days 17, 24 and 31 (indicated by black arrows in panel a).

SC injections of water-soluble Gem-PAAm prodrugs P9–P11 were carried out once a week during 3 consecutive weeks (*i.e.*, days 17, 24 and 31) at 60 mg per kg Gem equiv. dose (which corresponds to the MTD of Gemzar) and benchmarked against the IV-injection of the commercial formulation of Gem (Gemzar^IV^) at the same dose, in mice bearing Mia PaCa-2 pancreatic xenografts, which is a relevant model for Gem. The efficacy of the treatments was evaluated by following the evolution of tumor volume (Fig. 8a) and the survival rate (Fig. 8b).

Firstly, there were no adverse cutaneous reactions at the injection sites during treatment, demonstrating the absence of local toxicity of polymer prodrugs at such a dose as predicted by previous acute local toxicity study with P7^sc^ (see Fig. 6 and 7). Treatments with P9^SC^–P11^SC^ showed relatively similar tumor growth rates to Gemzar^IV^ up to 24 days after tumor induction (Fig. 8a), with an average tumor volume of ∼200 mm^3^. Subsequently, the tumor volume curves associated with P9^SC^–P11^SC^ began to slowly diverge from that of Gemzar^IV^ towards significantly lower average tumor volumes, indicating greater anticancer efficacy. In particular, at day 74, the tumor volumes associated with P9^SC^–P11^SC^ converged to a mean value of 1490 mm^3^, corresponding to a 22% tumor volume reduction compared with Gemzar^IV^ (1904 mm^3^). In addition, the evolution of the relative body weight loss in mice treated with Gem-PAAm prodrugs showed that the treatment was well tolerated, with mice losing no more than 10% on average of their body weight throughout the study (Fig. 8b). Remarkably, the SC injection of Gem-PAAm prodrugs also significantly increased the overall survival of mice. Indeed, 84 days after tumor induction, no mice survived in the Gemzar^IV^ group, whereas survival rates were 17, 36 and 33% for mice treated with P9^SC^, P10^SC^ and P11^SC^, respectively (Fig. 8c). The lower survival rate observed with P9^SC^ could be explained by the excessively short PAAm chain length, which led to a too rapid excretion of the prodrug, not allowing a significant release of Gem. In contrast, longer PAAm chain lengths induced a greater stealth effect and thus longer circulation times, allowing sustained release of Gem. Despite similar survival rates observed with P10^SC^ and P11^SC^, and a lower drug loading for P11^SC^, we believe that P11^SC^ is the best candidate as it gave a 100% survival rate for much longer than the other two prodrugs.

## 4. Conclusion

In this work, we have designed SC-injectable, water-soluble polymer prodrugs based on Gem as an irritant anticancer drug and PAAm as a highly water-soluble polymer. These polymer prodrugs were also made degradable by the insertion of ester groups in the main chain through rROP of BMDO with AAm during the “drug-initiated” synthesis. Not only the Gem-P(AAm-*co*-BMDO) copolymer prodrugs exhibited rapid degradation in a few days under physiological conditions, but they also showed tunable UCST properties depending on the BMDO content, allowing solubilization at body temperature after injection.

Sustained drug release was achieved in human serum over a week and *in vitro* assays on a pancreatic cancer cell line showed significant cytotoxicity of the polymer prodrugs. Degradable and non-degradable polymer prodrugs were easily injected under clinically relevant SC injection conditions, up to high doses. Importantly, SC injection of Gem-PAAm prodrugs and their degradable counterparts at high doses to mice did not induce local toxicity or even inflammation compared to free Gem, which engendered severe inflammation and even necrotic areas. This suggested that these prodrugs can be safely injected subcutaneously without the BMDO units and degradation products being toxic. Remarkably, the SC administration of Gem-PAAm prodrugs to mice bearing Mia Pa-Ca 2 tumor xenografts resulted in anticancer efficacy and survival rates superior to those of the commercial formulation of Gem (Gemzar®), injected intravenously.

Altogether, these results therefore successfully demonstrated the possibility of switching from IV administration of Gemzar® to SC administration of water-soluble, Gem-PAAm prodrugs. They also propose alternative polymer backbones that are degradable, non-cytotoxic, and do not induce any cutaneous toxicities *in vivo*, opening the path to designing polymeric systems with better body clearance outcomes. Finally, this research work argues in favor of the use of the “drug-initiated” method to design water-soluble polymer prodrugs for SC administration of vesicant/irritant anticancer drugs and pave the way for safer and less costly administration of chemotherapeutics.

## Author contributions

J. N. conceived and designed the research, and obtained funding; L.G designed the experiments; L. G., J. C., M. A. performed the experiments; A. B., C. Z., D. L., S. M. I., C. C. helped to perform the experiments; F. M. N. performed histology studies; L. G., J. N. wrote the manuscript. All authors contributed to the discussion of the results and to the revision of the manuscript.

## Conflicts of interest

The authors declare no competing interests.

## Supplementary Material

SC-OLF-D5SC02967H-s001

## Data Availability

Data will be made available on request.
