# Supplementary material for: Degradable water-soluble polymer prodrugs for subcutaneous delivery of irritant anticancer drugs
Source: Chem Sci. 2025 Jul 10;16(31):14323–41. doi: 10.1039/d5sc02967h (PMC12243153; doi:10.1039/d5sc02967h)
Supplement: SC-016-D5SC02967H-s001 [file SC-016-D5SC02967H-s001.pdf]

Supplementary Information (SI) for Chemical Science.
This journal is © The Royal Society of Chemistry 2025

Electronic Supplementary Information for

Degradable Water-Soluble Polymer Prodrugs for Subcutaneous Delivery of Irritant Anticancer Drugs

Léa Guerassimoff,[1] Jingming Cao,[1] Michaella Auguste,[1] Amaury Bossion,[1] Chen Zhu,[1] Dao Le,[1] Catherine Cailleau,[1] Safa Mohamed Ismail,[1] Françoise Mercier-Nomé,[2] Julien Nicolas[1]*

[1] Université Paris-Saclay, CNRS, Institut Galien Paris-Saclay, 91400 Orsay, France

[2] Université Paris-Saclay, IPSIT, INSERM UMR 996, 91400 Orsay, France

*To whom correspondence should be addressed.

E-mail: julien.nicolas@universite-paris-saclay.fr

Tel.: +33 1 80 00 60 81

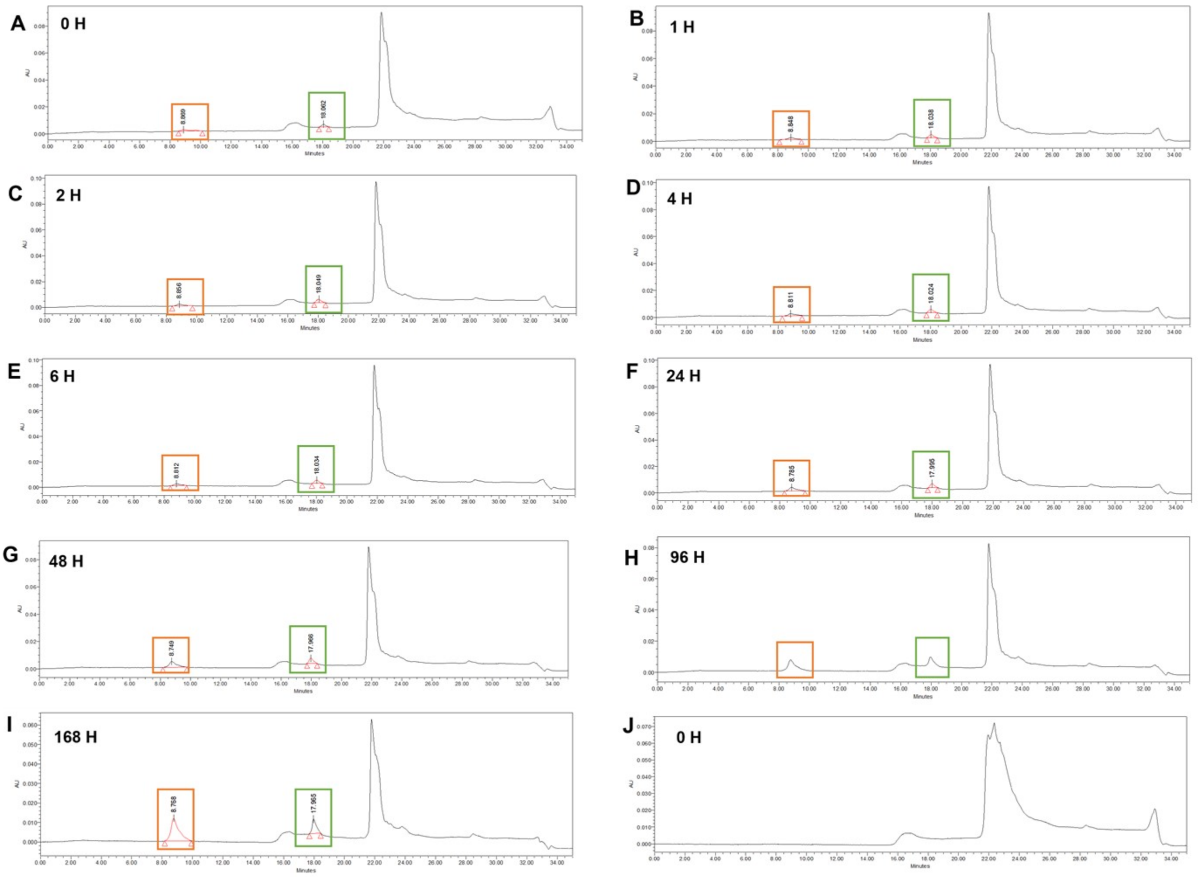

Figure S1. HPLC graphs of Gemcitabine release from Gem-P(AAm-*co*-BMDO) **P2** at 37 °C in MilliQ water after: (**A**) 0 h, (**B**) 1 h, (**C**) 2 h, (**D**) 4 h, (**E**) 6 h, (**F**) 24 h, (**G**) 48 h, (**H**) 96 h and (**I**) 168 h. The HPLC graph of MilliQ water alone is also provided at 0 h (**J**). The peaks corresponding to Gemcitabine and to Theophylline are highlighted by orange and green boxes, respectively.

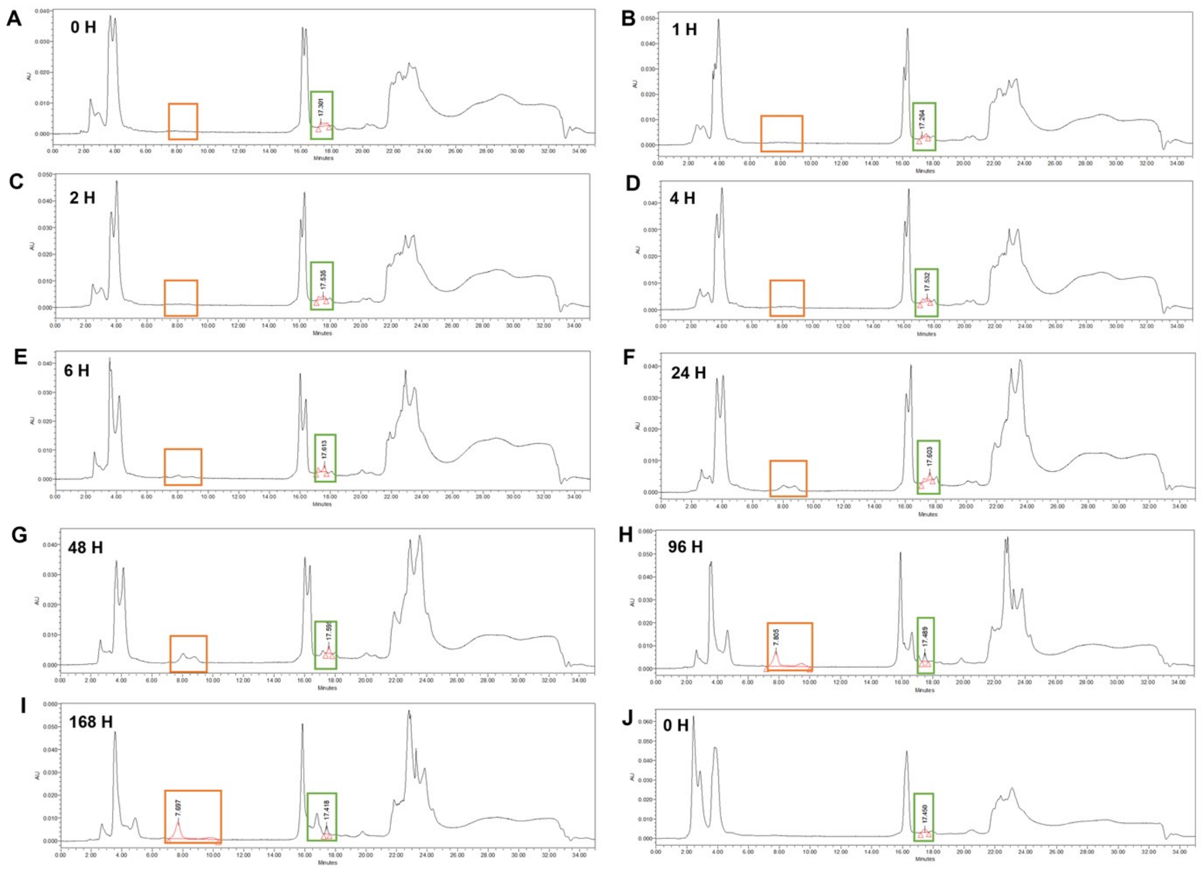

Figure S2. HPLC graphs of Gemcitabine release from Gem-P(AAm-*co*-BMDO) **P2** at 37 °C in Human serum after (**A**) 0 h, (**B**) 1 h, (**C**) 2 h, (**D**) 4 h, (**E**) 6 h, (**F**) 24 h, (**G**) 48 h, (**H**) 96 h and (**I**) 168 h. The HPLC graph of human serum alone with Theophylline standard at 0 h is also provided (**J**). The peaks corresponding to Gemcitabine and to Theophylline are highlighted by orange and green boxes, respectively.

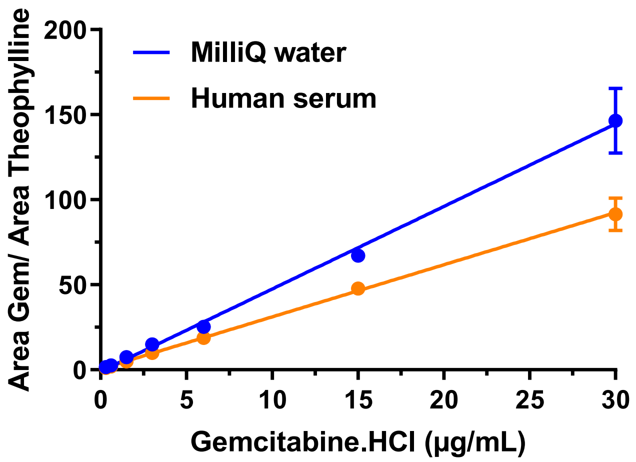

Figure S3. Calibration curves of Gemcitabine release in MilliQ water (blue curve) and in Human serum (orange curve) at 37 °C. The calculation of Gemcitabine release has been normalized using the peak area of Theophylline (10 µM) as standard (see experimental part section).

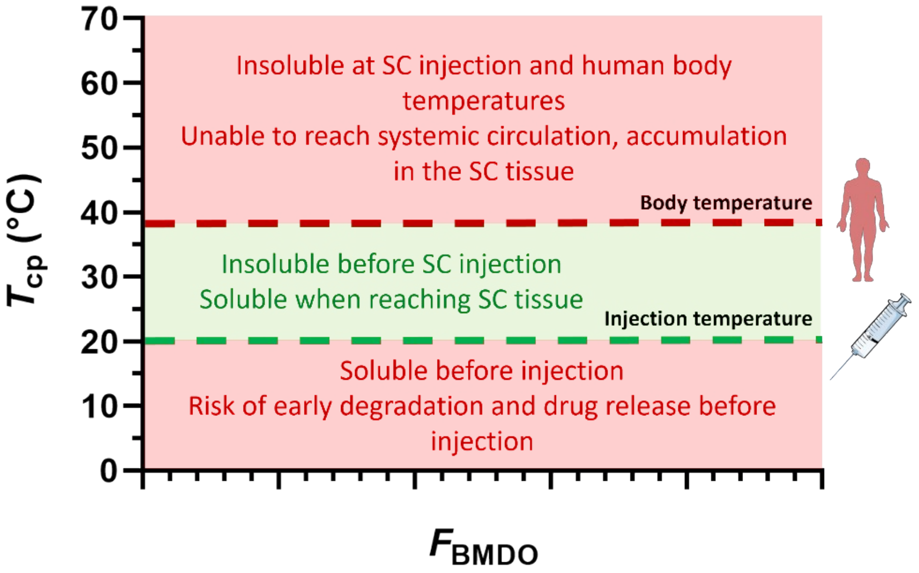

Figure S4. Rationale for the development of degradable, UCST copolymer prodrugs for the SC administration of anticancer drugs.

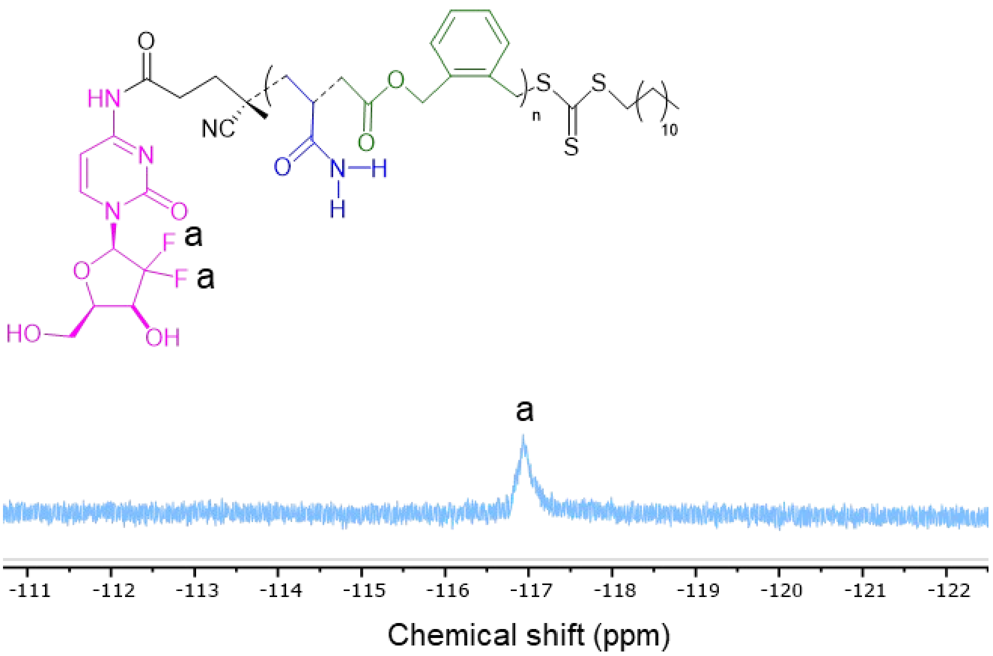

Figure S5. ^{19}F-NMR spectrum (400 MHz, DMSO-d_6) in the 111–122 ppm region of Gem-P(AAm-*co*-BMDO) **P2**.

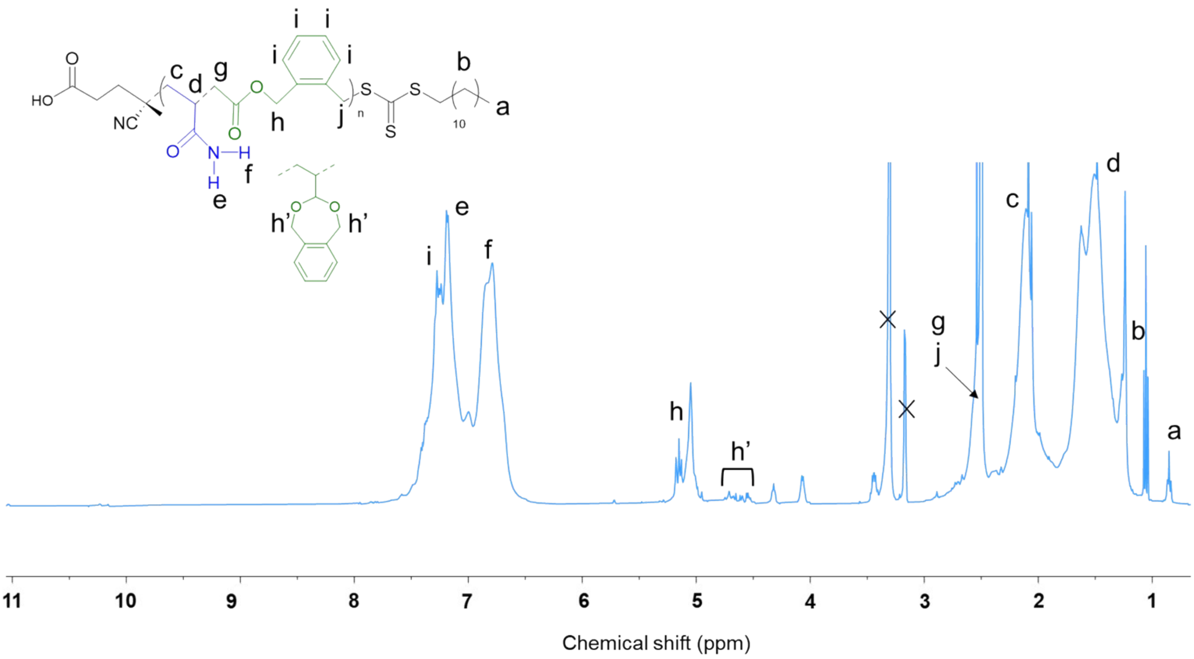

Figure S6. ^{1}H-NMR spectrum (400 MHz, DMSO-d_6) in the 0–11 ppm region of P(AAm-*co*-BMDO) **P5**.

Table S1. Calculation of the average number of AAm monomer units that follow each other before one BMDO unit is inserted ($\tilde{n}_{AAm}$) for copolymers **P0–P4**.

Entry	f_{AAm}	f_{BMDO}	$\tilde{n}_{AAm}$[a]
**P0**	0.50	0.50	~14
**P1**	0.55	0.45	~17
**P2**	0.56	0.44	~18
**P3**	0.57	0.43	~18
**P4**	0.60	0.40	~21

[a] Determined by: $\tilde{n}_{AAm} = (r_{AAm} \times f_{AAm} + f_{BMDO}) / f_{BMDO}$, with r_{AAm} the reactivity ratio of AAm (13.02) and f_{AAm} and f_{BMDO} the initial molar fractions in AAm and BMDO, respectively.

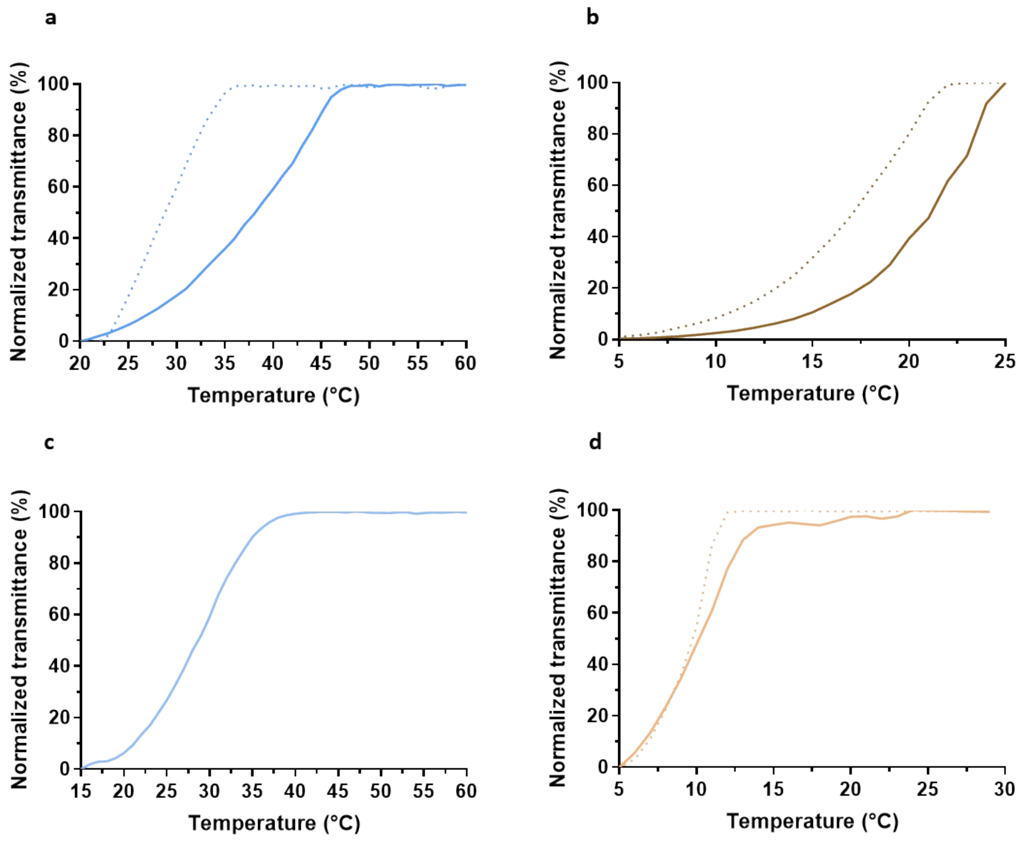

Figure S7. Variation of the solution transmittance with temperature of: **a**, Gem-P(AAm-*co*-BMDO) **P2**; **b**, Gem-P(AAm-*co*-BMDO) **P3**, and **c**, P(AAm-*co*-BMDO) **P5** and **d**, P(AAm-*co*-BMDO) **P6** upon heating (solid lines) and cooling (dotted lines) at 1 °C.min^{-1} in MilliQ water at 1.23 mg.mL^{-1} (i.e., concentration used for MTT assays). No transition appeared upon cooling for **P5**.

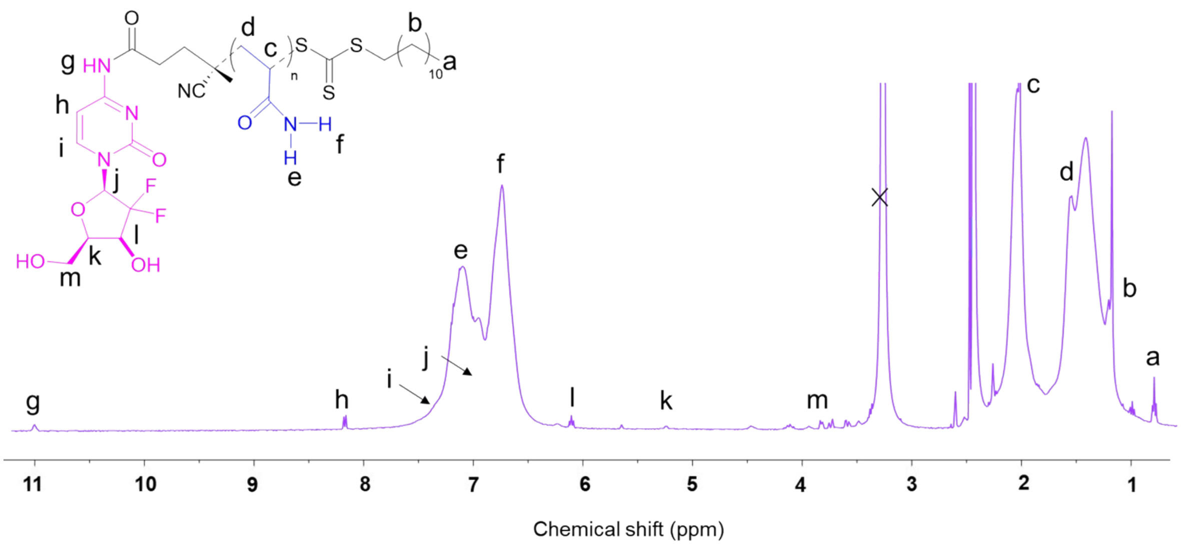

Figure S8. ^{1}H-NMR spectrum (400 MHz, DMSO-d_6) in the 1–11 ppm region of Gem-PAAm **P7**.

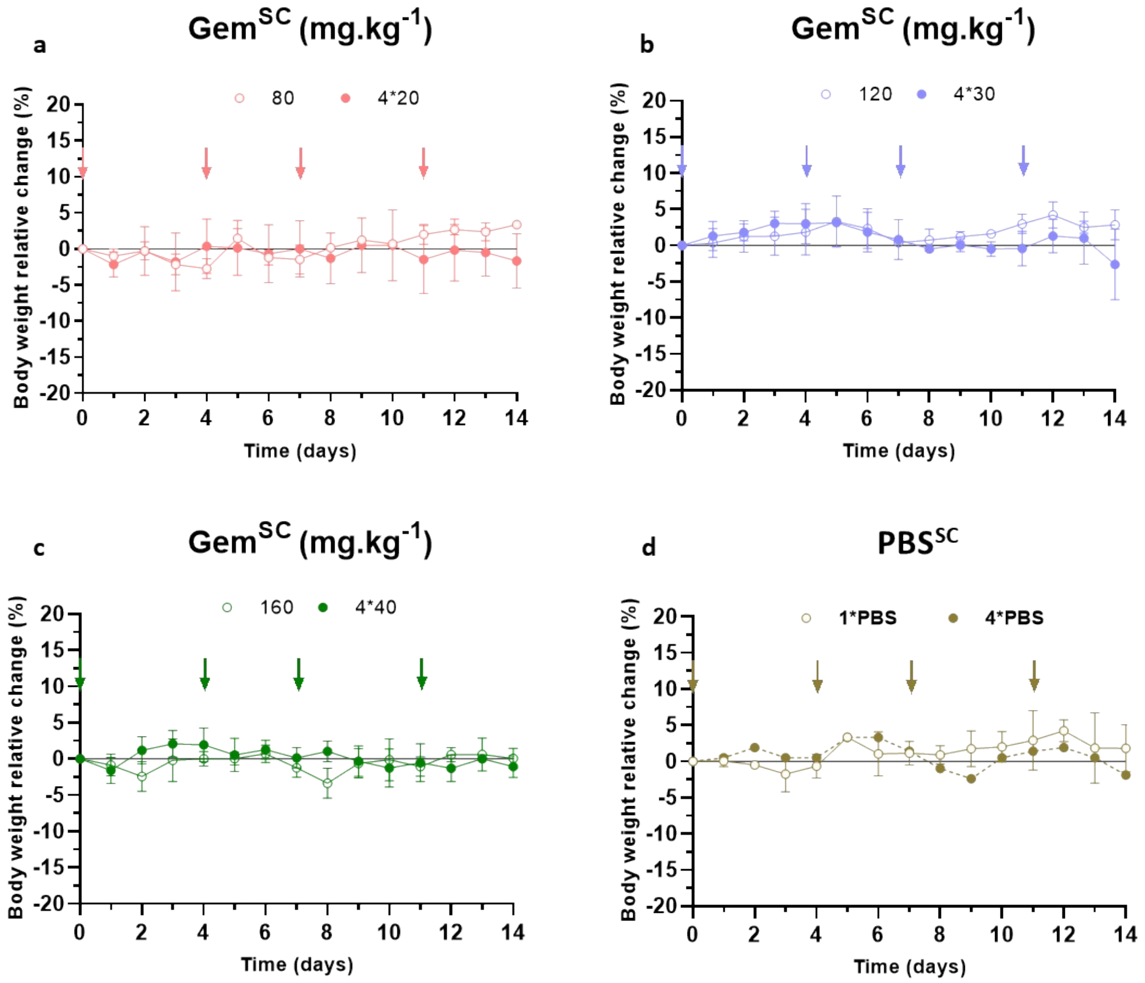

Figure S9. Relative body weight changes of mice over two weeks after SC injection of Gem (Gem^{SC}) at different doses: **a**, 80; **b**, 120; **c**, 160 $mg.kg^{-1}$ and of **d** PBS. Two protocols of injection were tested: (i) a single injection of the dose at day 0 and (ii) 4 injections of a quarter of dose each at days 0, 4, 7 and 11, indicated by arrows.

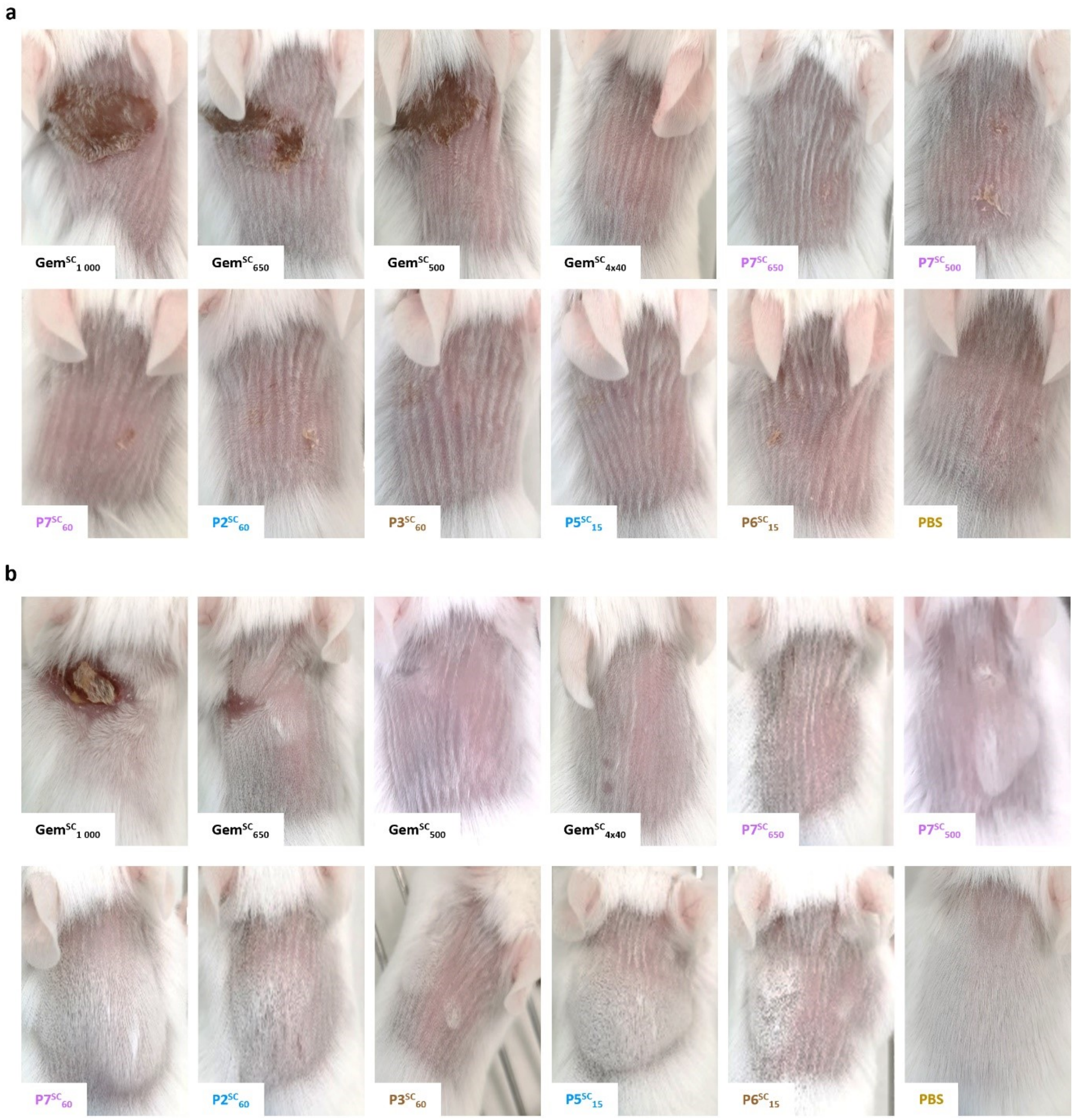

Figure S10. Representative pictures of mice (n = 3): **a**, the first day and **b**, 14 days after SC injection of Gem at 1 000, 650, 500 mg.kg^{-1} (single injection) and 160 mg.kg^{-1} (after 4 injections), Gem-PAAm **P7** at 650, 500 and 60 mg.kg^{-1}, Gem-P(AAm-*co*-BMDO) **P2** and **P3** at 60 mg.kg^{-1}, drug-free P(AAm-*co*-BMDO) **P5** and **P6** at 15 mg.kg^{-1} (equiv. Gem) and PBS.

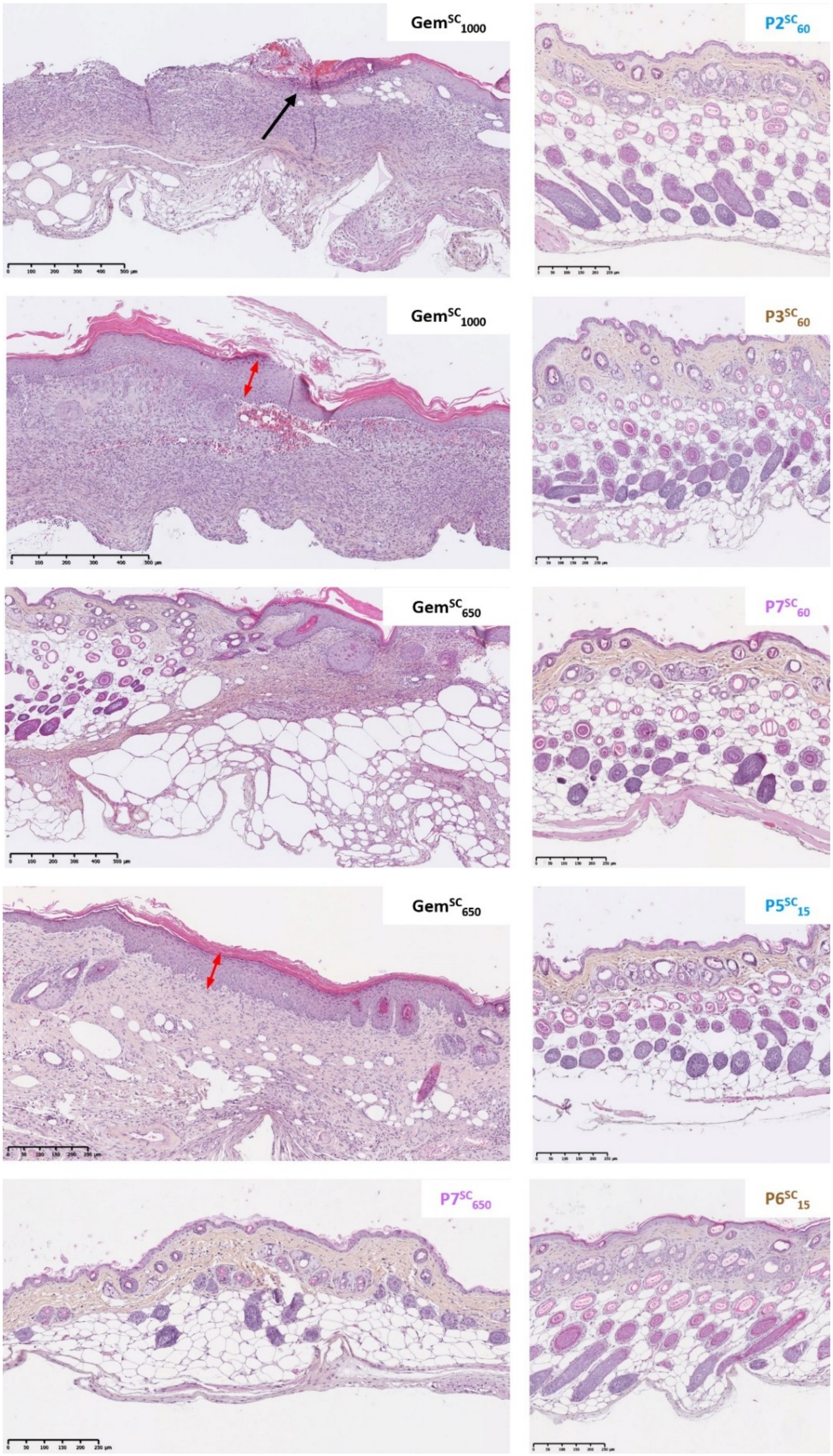

Figure S11. Representative HES-stained sections of skin samples after SC injection of Gem (650 and 1 000 mg.kg^{-1}), Gem-P(AAm-*co*-BMDO) **P2** and **P3** at 60 mg.kg^{-1}, Gem-PAAm **P7** at 650 and 60 mg.kg^{-1}, and drug-free P(AAm-*co*-BMDO) **P5** and **P6** at 15 mg.kg^{-1}(equiv. Gem). Red arrows indicate hyperplasia of the epidermis and the black arrow underlines a necrosis zone.

Table S2. Histopathological scores (H-scores) evaluated by cutaneous/SC degenerative and necrotic changes and by inflammation.

SC treatment ($mg.mL^{-1}$ equiv. Gem)	Histopathological changes	Cutaneous/SC degenerative and necrotic changes[a]	Inflammation[a]
**PBS**	No significant histopathological lesion	0	0
**Gem (500)**	Large epidermal hyperplasia with few granulomatous foci in the dermis	0	1
**Gem (650)**	Large epidermal hyperplasia with few granulomatous foci in the dermis	0	1.5
**Gem (1000)**	Focal deep hypodermal muscular necrosis associated with few granulomatous foci in the deep hypodermis	1.5	3
**P7 (60)**	No significant histopathological lesion	0	0
**P7 (500)**	No significant histopathological lesion	0	0
**P7 (650)**	No significant histopathological lesion	0	0
**P2 (60)**	No significant histopathological lesion except small epidermal hyperplasia	0	0
**P3 (60)**	No significant histopathological lesion except small epidermal hyperplasia	0	0
**P4 (15)[b]**	No significant histopathological lesion except small epidermal hyperplasia	0	0
**P5 (15)[b]**	No significant histopathological lesion except small epidermal hyperplasia	0	0

[a]Semi-quantitative score from 0 (no change) to 3 (marked change); [b]equiv.Gem for copolymer prodrug counterparts.

`

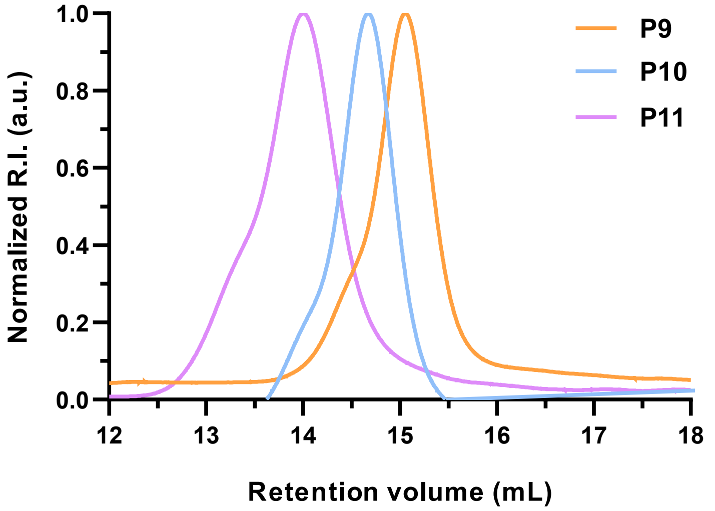

Figure S12. SEC chromatograms in DMSO of Gem-PAAm polymer prodrugs of three different molecular weights: 6180 (**P9**), 12 260 (**P10**) and 21 340 (**P11**) g.mol^{-1} (Table 1) used for anticancer efficacy studies.
